# Application of Amino Acids in the Structural Modification of Natural Products: A Review

**DOI:** 10.3389/fchem.2021.650569

**Published:** 2021-04-29

**Authors:** Qian Xu, Hao Deng, Xiaoting Li, Zhe-Shan Quan

**Affiliations:** ^1^Key Laboratory of Natural Medicines of the Changbai Mountain, Affiliated Ministry of Education, College of Pharmacy, Yanbian University, Jilin, China; ^2^Department of Pharmaceutical Analysis, Wuya College of Innovation, Shenyang Pharmaceutical University, Shenyang, China

**Keywords:** derivatives, research progress, structure modification, natural products, amino acids

## Abstract

Natural products and their derivatives are important sources for drug discovery; however, they usually have poor solubility and low activity and require structural modification. Amino acids are highly soluble in water and have a wide range of activities. The introduction of amino acids into natural products is expected to improve the performance of these products and minimize their adverse effects. Therefore, this review summarizes the application of amino acids in the structural modification of natural products and provides a theoretical basis for the structural modification of natural products in the future. The articles were divided into six types based on the backbone structures of the natural products, and the related applications of amino acids in the structural modification of natural products were discussed in detail.

## Introduction

Amino acids are the basic units of proteins and the primary substance supporting biological life activities. They are closely related to the activities of a living body, playing very important roles. They are essential organic molecules in any organism. The amino acid chemical structure contains an amino group (-NH_2_), a carboxylic acid group (-COOH), and a unique side chain on the carbon that connects the amino group to the carboxyl group. Amino acids have many special physiological functions, playing roles in protein synthesis, metabolism, body development, osmotic pressure stability, and neurotransmission (Wu, 2009).

In addition to these functions, amino acids are also widely used in the food industry. For example, the main raw material of the sweetener aspartame is *L*-phenylalanine (Liu et al., 2019). Naturally occurring amino acids can be used to enhance the flavor of food and enhance nutrition (Ikeda, 2003). Additionally, amino acids are widely used in the medical and healthcare industries (Carubelli et al., 2015; Nitz et al., 2019). They also have a wide range of pharmacological activities, such as antitumor (Barnham et al., 1996), anti-HIV (Chen A. et al., 2006), and anti-fatigue (You et al., 2011) effects; in addition, they are used to cure chronic liver diseases (Kawaguchi et al., 2011).

The structures of amino acids are simple and diverse, and their pharmacological activities are extensive. These features are commonly employed in drug synthesis and structural modification. It has been reported that after a compound is combined with an amino acid, the pharmacological activity of the compound is enhanced (Ma et al., 2007; Zhang et al., 2008), water solubility is improved (Drag-Zalesinska et al., 2009; Kim et al., 2009), and cytotoxicity is reduced (Anbharasi et al., 2010).

**Figure 1:**
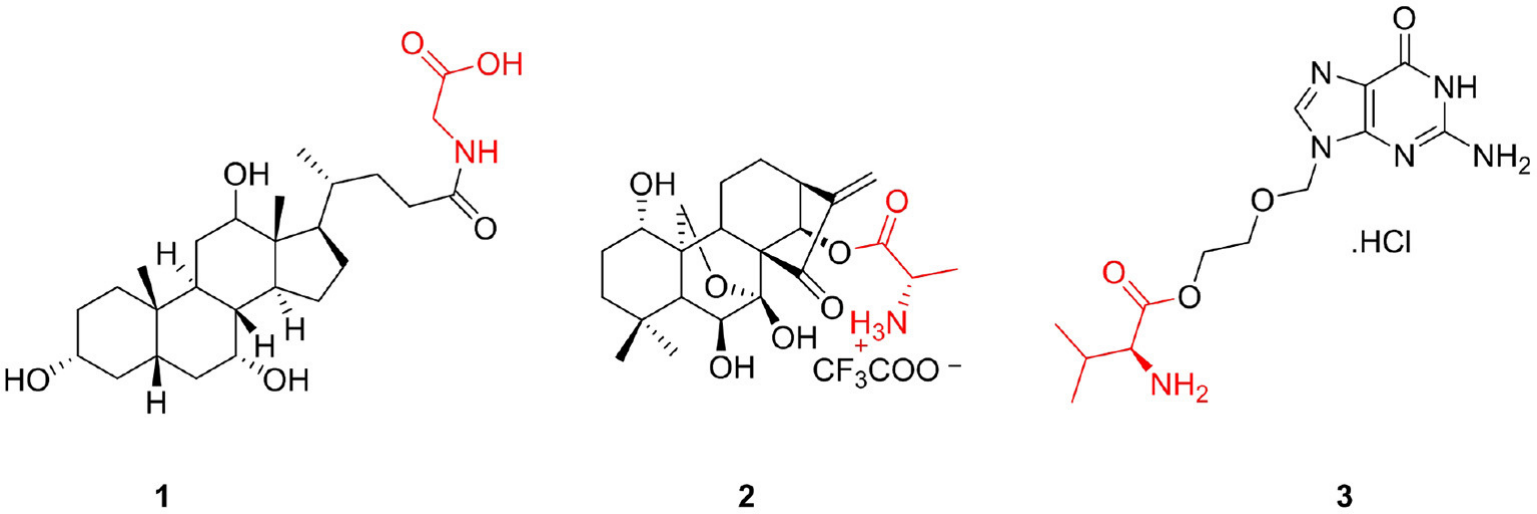
Successful cases of natural product modification with amino acids.

Natural products and their molecular frameworks are valuable sources of medicinal chemistry and drug discovery (Lee, 2004). In the past decades, drugs that directly or indirectly replace derivatives and analogs of natural products have played important roles in the fight against diseases (Rodrigues et al., 2016). Although the biological activities of natural products are extensive, they are not strong and often have the disadvantages of low bioavailability and poor solubility (Shi et al., 2010). Therefore, to enhance their physchem properties and ADME, it is important to make necessary structural modifications to these products. Interestingly, amino acids can improve these shortcomings, and therefore, they have become the preferred natural product structural modifiers. Many natural products have shown improved biological activity and physicochemical properties following the introduction of amino acids (Ma et al., 2000; Brady et al., 2002), and successful modification results have been obtained. Glycolic acid (**1**) is the product of bile acid linked to glycine that shows anticancer activity by targeting the pump resistance-related and non-pump resistance-related pathways (Lo et al., 2008); it is currently undergoing Phase III clinical studies (Figure 1). Furthermore, HAO472 (**2**) is a TFA salt compound composed of oridonin and alanine that possesses potential activity to treat acute myelogenous leukemia and is presently undergoing a Phase I clinical trial in China conducted by Hengrui Medicine Co. Ltd. (Figure 1) (Sun et al., 2014). Additionally, valaciclovir hydrochloride (**3**), an adenine linked to valine via a glycol linker, is an antiviral drug that has been marketed for the treatment of herpes simplex, herpes zoster, and herpes B (Figure 1) (LaVail et al., 2003).

Natural products constitute a potential starting point for new drug design; thus, this review summarizes the structural modification of natural products by amino acid as has been reported between 2010 and 2020. Relevant articles were retrieved from various databases and analyzed. This review is expected to provide a theoretical basis for the structural modification of natural products in the future.

## Application of Amino Acids in the Structural Modification of Natural Products

Numerous natural products exist; thus, to facilitate the classification and management of literature, this review classifies all relevant articles into six categories based on the lead framework, including phenylpropanoids, quinones, flavonoids, terpenes, alkaloids, and polyphenols.

### Phenylpropanoids

#### Cinnamic Acid

**Figure 2:**
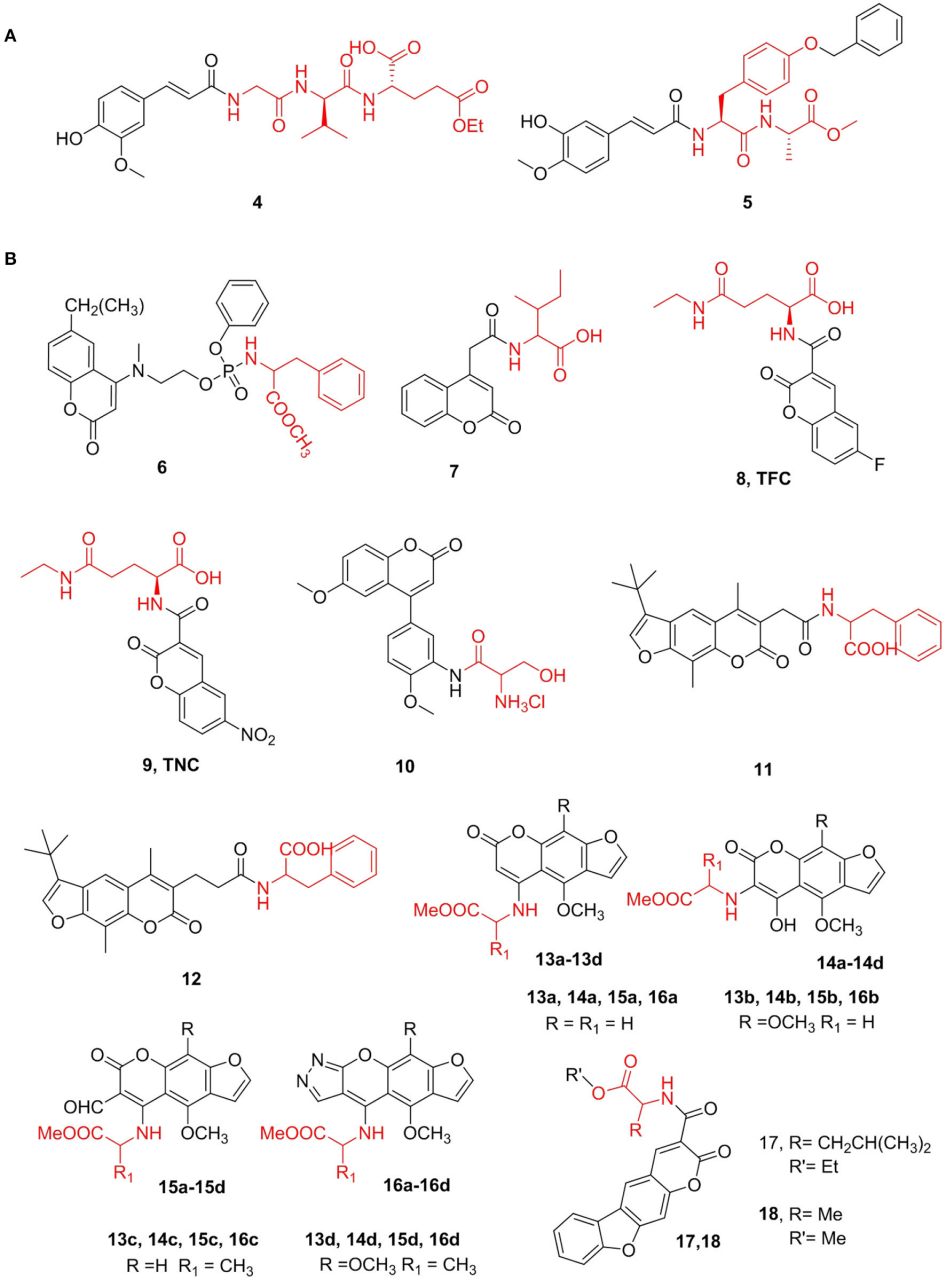
**(A)** Cinnamic acid derivatives. **(B)** Coumarins amino acid derivatives.

Ferulic and isoferulic acids are cinnamic acid compounds containing phenolic hydroxyl groups and exert various biological effects. Gobec et al. (2018) discovered compound **4**, a tripeptide derivative of isoferulic acid, which is presently the most potent desmuramylpeptide NOD2 agonist, and the EC_50_ value for NOD2 is 46 nM. Compound **4** can cooperate with lipopolysaccharide to increase the release of pro-inflammatory cytokines in human peripheral blood mononuclear cells, and it induces ovalbumin-specific IgG titers in a mouse model of adjuvant. Further, Hamed et al. (2020) synthesized a series of *N*-feruloyl dipeptides and conducted in-depth studies of these compounds as potential anticancer agents against 10 cancer cell lines from different tissue origins. Based on this, compound **5** showed the strongest cytotoxic activity for all cell lines with IC_50_ values ranging from 2.1 to 7.9 μM, and its activity was stronger than that of ferulic acid (IC_50_ > 30 μM) (Figure 2A).

#### Coumarin

Coumarin compounds are an important class of natural heterocyclic compounds with antioxidant, antibacterial, anti-inflammatory, and anticancer properties. Since these compounds have a wide range of pharmacological activities and simple chemical structures, they are favored by many medicinal chemistry researchers. Ji et al. (2016) synthesized novel coumarin phosphoramide derivatives and screened for chitin synthase inhibitory and antibacterial activities *in vitro*. It was found that most of these compounds have a good inhibitory effect on chitin synthase. Among them, compound **6**, with an IC_50_ value of 0.08 mM, was shown to be the strongest chitin synthase inhibitor. Additionally, antifungal experiments have shown that these compounds have moderate or even excellent antibacterial activity against common clinical pathogens. Further, Patagar et al. (2019) designed and synthesized substituted coumarin-4-acetyl amino acids and determined their inhibitory activity against DHODH, a dual-target compound for malaria and cancer. The DHODH inhibitory activity of the newly synthesized compound was then determined. Compared with the standard product, compound **7** showed obviously the highest 70.6% inhibition of DHODH at 0.5 μM, and other compounds with terminal COOH showed moderate inhibition (Figure 2B).

Zhang et al. (2014) synthesized ethyl 6-fluorocoumarin-3-carboxy-*L*-theanine (**8**) and ethyl 6-nitrocoumarin-3-carboxy-*L*-theanine (**9**). These two compounds enhance the activities of the anticancer drugs cytarabine, vincristine, and methotrexate in inhibiting the growth of lung cancer cells and have no toxic effects on normal human embryonic lung fibroblasts and peripheral blood lymphocytes. TFC (IC_50__LLC_ = 0.125 mM and IC_50A549_ = 0.99 mM) and TNC (IC_50__LLC_ = 0.09 mM and IC_50A549_ = 0.064 mM) have shown strong inhibitory effects on the growth of highly metastatic LCC and A549 tumors in tumor-bearing mice and are non-toxic to mice. Additionally, Malysheva et al. (2014) obtained novel 4-arylcoumarin derivatives and found that water-soluble salts (**10**) show considerable cytotoxicity against the HaCaT cell line (IC_50_ = 0.07 μM) (Figure 2B).

In addition to the amino acids in furanocoumarin, all synthetic compounds have been tested as EGFR and VEGFR-2 kinase inhibitors. The EGFR enzyme inhibition IC_50_ values of all compounds range from 20.2 to 57.6 nM, while the EGFR-2 enzyme inhibition (IC_50_) ranges from 1.2 to 58.9 nM. In general, compound **16d** had the strongest inhibitory effect on the two targets. Francisco et al. (2012) synthesized two new tetracyclic benzofuranocoumarin (benzopsoralen) analogs (compounds **17** and **18**) and found that they significantly inhibited the proliferation of MDA MB 231, HeLa, and TCC-SUP with GI_50_ of three tumor cell lines is between 0.043 and 0.205 μM, which is thought to be mainly linked to the inhibition of CYP2A6 (Figure 2B).

#### Lignans

Lignans are a kind of natural product formed by the oxidative polymerization of phenylpropanoids, containing two molecules of C6–C3 structure. Podophyllotoxin (PPT), a natural antimitotic product isolated from *Podophyllum peltatum* L (Imbert, 1998), exhibits various interesting biological activities, such as antitumor and antiviral activities (Garcia and Azambuja, 2004). However, PPT displays many side effects, including damage to normal tissues, leading to the development of lower cytotoxicity and higher efficiency PPT derivatives.

**Figure 3:**
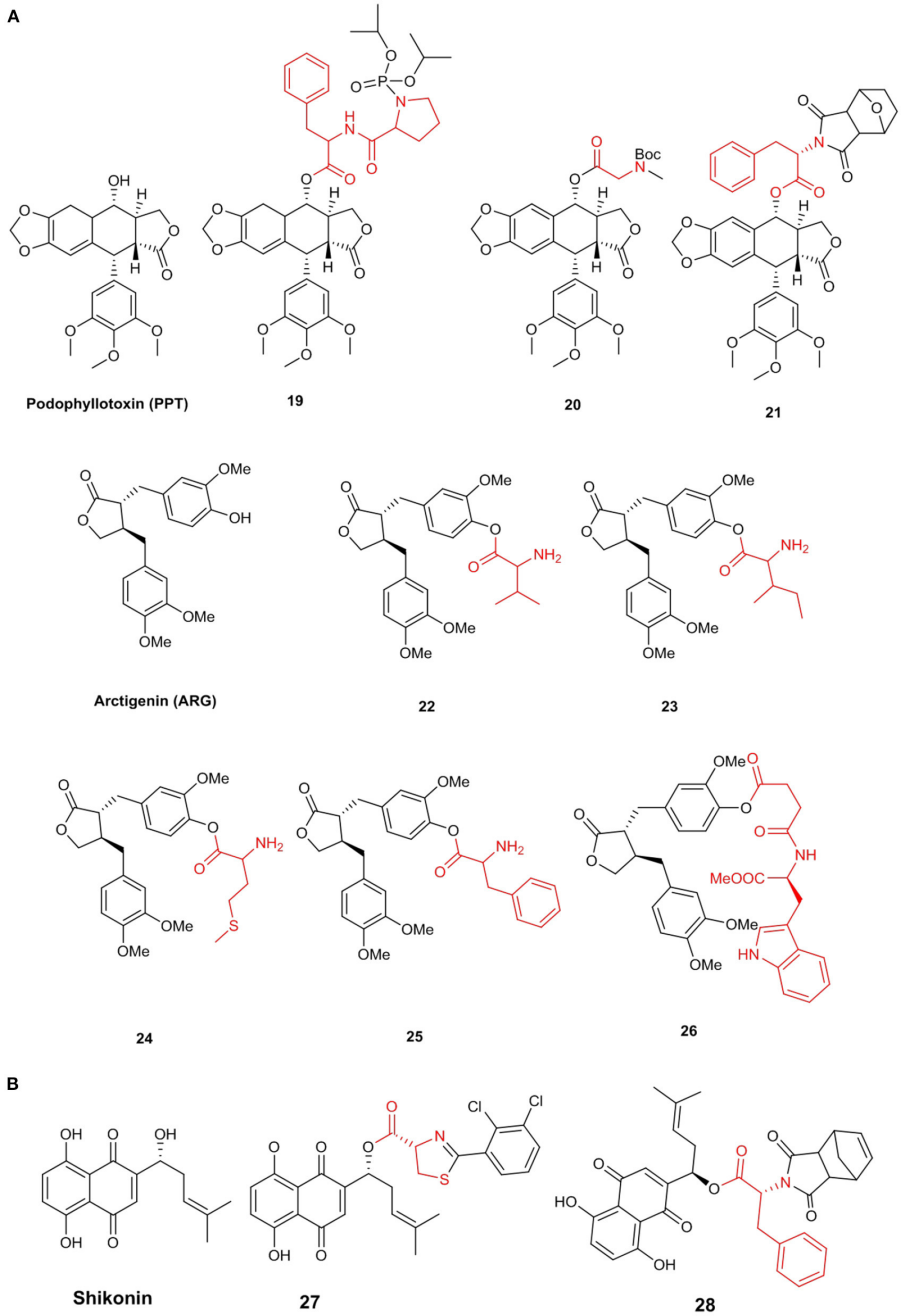
**(A)** Lignans amino acid derivatives. **(B)** Shikonin amino acid derivatives.

Hu et al. (2011) synthesized a series of novel compounds at the C-4 of PPT and evaluated their cytotoxicity in the K562 cell line *in vitro*. Compound **19** exhibited better cytotoxicity (IC_50_ = 2.1 μM) than those of PPT and etoposide, indicating that the antitumor effect might be because of the induction of apoptosis. Further, Wu et al. (2018) synthesized a series of PPT derivatives and tested their cytotoxicity in several cancer and L02 cell lines. Interestingly, some *N*-Boc amino acid target demonstrated high selectivity, especially compound **20**, which showed a high degree of selectivity for both cancer and normal cells. The IC_50_ values of compound 20 in A549, MCF-7, HepG2, and L02 cells were 9.5, 132.6, 96.4, and 160.2 nM, respectively, showing low cytotoxicity and high selectivity. Moreover, compound **20** induced apoptosis in A549 cells and prevented the transition of these cells from the S phase to the G2 phase; however, it had no significant effect on L02 cells. Han et al. (2016) screened an effective podophyllotoxin derivative, namely **21** (IC_50_ in MCF-7 cell line = 0.9 μM), with low cytotoxicity to non-cancer cells, including human embryonic kidney cells (293T) (IC_50_ = 54.4 μM). Additionally, this derivative can cause a significant cell cycle arrest at the G2/M phase and induce MCF-7 cell apoptosis more than PPT. Moreover, the expression of cell cycle protein CDK1 was upregulated, while a protein required for mitotic initiation, Cyclin B1, was downregulated. This result showed that **21** was an effective tubulin polymerization inhibitor, and its effect is equivalent to that of positive control colchicine (Figure 3A).

Arctigenin (ARG) is a lignan-class compound isolated from *Arctium lappa* L. According to reports, it has a variety of biological activities, such as antitumor, anti-inflammatory, and antiviral activities. Although ARG has a variety of pharmacological activities *in vitro*, it is insoluble in the human body and thus cannot be absorbed, limiting its clinical application. Therefore, it is very important to use chemical or biological methods to modify ARG into derivatives with better solubility and higher bioavailability (Figure 3A).

Cai et al. (2016) synthesized a series of ARG amino acid derivatives, all of which have better solubility and nitrite elimination ability than those of ARG. This compound and its two amino acid derivatives without a Boc protection group (**22**, **23**) were selected for *in vivo* antitumor activity evaluation. In H22 tumor-bearing mice, the tumor inhibition rates of compounds **22** and **23** were 69.3 and 43.6% at a dosage of 40 mg/kg, respectively, which was significantly higher than that of ARG. Besides, compared with the positive group, these compounds caused less damage to the liver, kidney, and immune organs of mice. Additionally, they synthesized five other amino acid derivatives (Cai et al., 2018) and measured the antitumor activity of these compounds. The inhibition rates of compounds **24** and **25** were 55.9 and 51.4% at a dosage of 40 mg/kg, respectively, which were twice than that of ARG. Moreover, the compounds were found to reduce damage to internal organs. Further, Zhang H. B. et al. (2018) prepared a series of ARG amino acid derivatives, measured their anti-*Toxoplasma gondii* activities *in vitro*, and calculated their selectivity indices. These derivatives showed greater selectivity indices than that of ARG and good anti-*Toxoplasma gondii* activities. The selectivity index of compound **26** was 3.2, which showed low toxicity to host cells and high anti-*Toxoplasma* activity (Figure 3A).

### Quinones

They are widely distributed in nature and are divided into four types: benzoquinone, naphthoquinone, phenanthrenequinone, and anthraquinone.

#### Naphthoquinone

Shikonin is an active naphthoquinone compound isolated from the root of the traditional Chinese Medicine *Lithospermum erythrorhizon* Sieb. et Zucc. (Chen et al., 2002). Shikonin has received extensive attention from medicinal chemistry researchers due to its particularly good anticancer activity (Lin et al., 2013). However, the side effects and cytotoxicity of shikonin limited its application as a new clinical anticancer drug (Cui et al., 2008). Lin et al. (2015) synthesized a series of shikonin derivatives containing a *L*-cysteine residue and determined their anticancer activities. Among them, compound **27** showed better antiproliferative activity with IC_50_ = 3.1_μM in HeLa cells than that of shikonin (IC_50_ = 5.8 μM). In addition, this compound may cause cell cycle arrest in the G2/M phase, lead to apoptosis, reduce the adhesion ability of HeLa cells, and bind well to tubulin at the paclitaxel binding site, resulting in tubulin polymerization and destruction mitotic. Further, Baloch et al. (2015) synthesized a series of other shikonin derivatives and evaluated their antiproliferative activities against five cancer cell lines, including A549, HeLa, MCF-7, HepG2, and BGC, with IC_50_ values ranging from 1.3 to 18.5 μM. Compared to shikonin itself and other compounds, compound **28** showed a better antiproliferative effect against all cancer cell lines and could induce apoptosis in a dose- and time-dependent manner, as well as arrest the cell cycle in the G2/M phase (Figure 3B).

#### Anthraquinone

**Figure 4:**
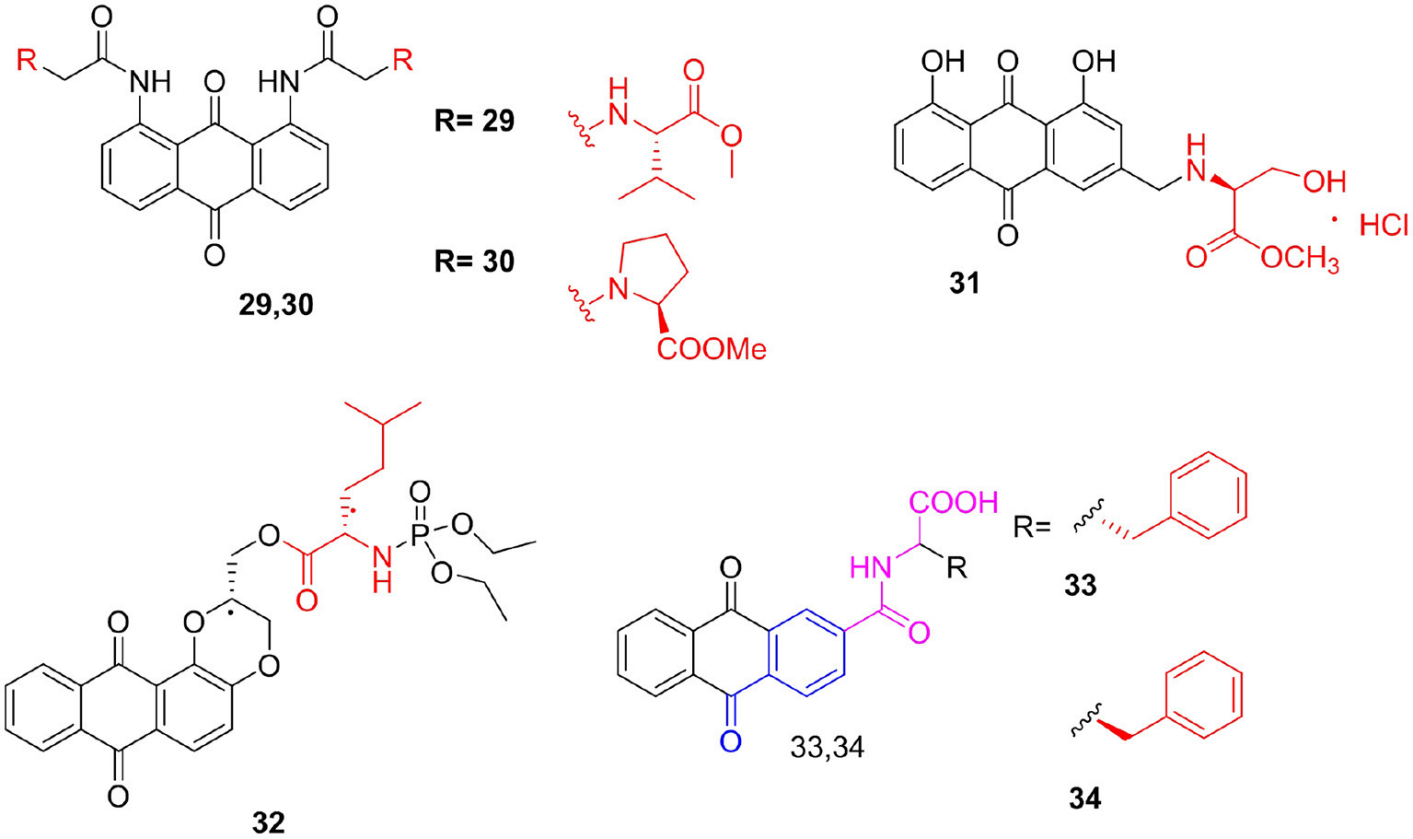
Anthraquinones amino acid derivatives.

Anthraquinone is currently the most important quinone compound with various biological activities, such as purgative, antibacterial, and anticancer activities. Since anthraquinone compounds have a polyaromatic ring-condensed structure, their solubility is extremely poor (<0.1 g/100 mL). Lee et al. (2012) synthesized a series of symmetrically dispersed 1,8-diamidoanthraquinones, evaluated their antiproliferative and telomerase inhibitory effects via a TRAP assay, and measured *h*TERT expression using SEAP. In particular, among all derivatives, compounds **29** and **30**, which are linked to valine and proline methyl esters, respectively, showed the most potent *h*TERT repressing activities, with IC_50_ both <10 μM. Aloe-emodin is a type of hydroxyanthraquinone found in aloe leaves (Pecere et al., 2000). Thimmegowda et al. (2015) focused on improving the water solubility of aloe-emodin and synthesized a series of amino acid derivatives, hoping to improve the efficacy of aloe-emodin. They found that the antitumor activity of derivative **31** against HepG2 (IC_50_ = 4.79 μM) and NCI-H460 (IC_50_ = 19.0 μM) tumor cell lines was better than that of aloe-emodin, which may be due to the increase in the number of hydroxyl groups and oxygen atoms of fatty acid esters. These groups and atoms increase solubility in water through hydrogen bonds and increase cell communication. Furthermore, Huang et al. (2017) synthesized the phospholipid amino acid alizarin and conducted an *in vitro* study on its inhibitory effect against tumor cell proliferation. All newly synthesized derivatives showed high cytotoxicity compared with alizarin and low cytotoxicity against the human normal liver HL-7702 cell line. Among them, compound **32** had the strongest killing effect on SK-OV-3 cells, with IC_50_ = 7.1 μM, slightly lower than that of doxorubicin. The anticancer activity of this compound depended on the apoptotic death of cancer cells by regulating members of the Bcl-2 family and arrest of the SK-OV-3 cell cycle in the G2 phase. Furthermore, Zhang T. J. et al. (2018) designed and synthesized a series of N-(9,10-anthraquinone-2-carbonyl) amino acid derivatives as novel xanthine oxidase inhibitors by mimicking the compound febuxostat. Among them, *L*-phenylalanine derivative **33** (IC_50_ = 3.0 μM) and *D*-phenylalanine derivative **34** (IC_50_ = 2.9 μM) demonstrated the strongest effect, and both were better than allopurinol (IC_50_ = 8.1 μM). Compared with the *L*-enantiomer, *D*-amino acid derivatives have the same or higher effect (Figure 4).

### Flavonoids

The basic nucleus of flavonoids is 2-phenylchromone. Flavonoids are widespread in nature and high in quantity in some plants and berries. They can be divided into flavones, isoflavones, chalcones, and other flavonoids according to their structure. These compounds, similar to the above-mentioned anthraquinones, have low solubility due to their long conjugated structures.

#### Flavones

**Figure 5:**
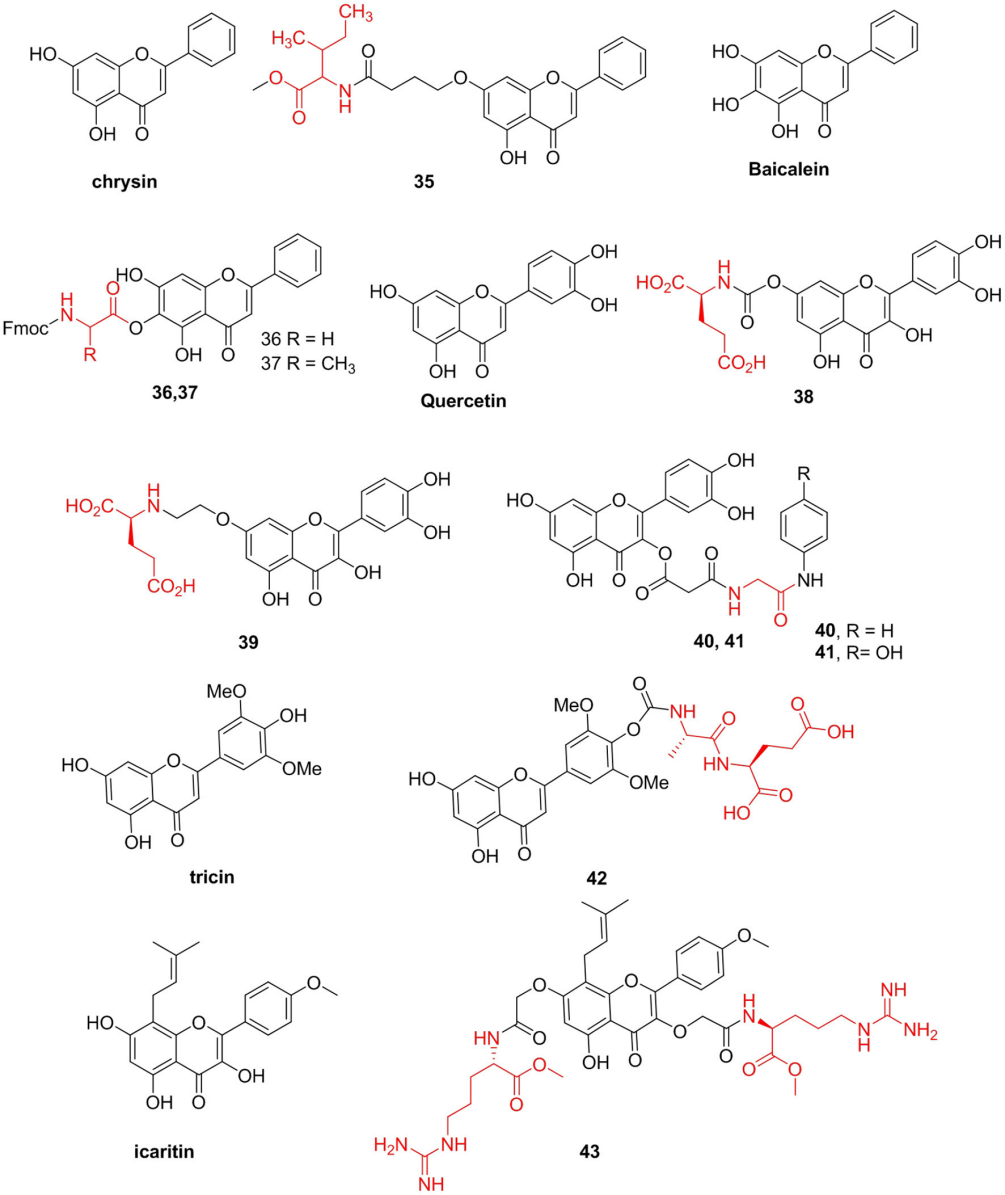
Flavonoids amino acid derivatives.

Song X. et al. (2014) prepared a series of chrysin-like amino acid derivatives and measured their antiproliferative activities in gastric cancer MGC-803 cells. They found that compound **35** (IC_50_ = 3.8 μM) had the strongest inhibitory effect on the growth of MGC-803 cells. This compound induced apoptosis of MGC-803 cells in a dose-dependent manner, increasing Bax and reducing Bcl-xl protein expressions. Baicalein, a natural flavonoid isolated from *Scutellaria baicalensis* Georgi (Lamiaceae), has been shown to have multiple biological activities. However, this flavonoid has poor solubility and low bioavailability, which limits its clinical application. Li et al. (2013) prepared baicalein amino acid derivatives and tested their *in vitro* cytotoxic activities. Compounds **36** (IC_50_ = 9.6 μM) and **37** (IC_50_ = 13.5 μM) showed significant increases in cytotoxicity compared with baicalein (IC_50_ = 40.2 μM) in HepG2 cells (Figure 5).

Furthermore, Kim et al. (2014) prepared quercetin–amino acid derivatives and evaluated their MDR-modulatory effects. A quercetin–glutamic acid derivative **38**, with IC_50_ values of 0.8–0.9 μM, was as effective as verapamil in reversing MDR and sensitizing MDR MES-SA/Dx5 cells to various anticancer drugs. The MDR reversal activity of this derivative is due to its hydrolyzable property. Additionally, there is evidence that the intact quercetin–glutamic acid derivative has stronger MDR-reversal activity than that of quercetin. Kim et al. (2017) synthesized quercetin derivatives with a glutamic acid attached at the 7-O position via a non-hydrolyzable linker. Derivative **39** showed significantly higher activities than those of quercetin. Compared with that of quercetin (1.9-folds), the structurally modified quercetin derivative (22.3-folds) with a non-cleavable linker showed significantly improved MDR-reversal activities. However, the quercetin derivatives were not as effective as verapamil in Pgp inhibition, thereby reversing MDR (Figure 5).

Furthermore, the natural product simocyclinone D8 (SD8) inhibits DNA gyrase via a unique mechanism. Verghese et al. (2013) synthesized flavone-based analogs inspired by the complex compound SD8. However, these analogs do not act as catalytic inhibitors, as SD8 does. Analogs **40** (IC_50_ = 48.6 μM) and **41** (IC_50_ = 84.7 μM) were determined to be DNA intercalators and topoisomerase poisons.

Tricin has demonstrated diverse biological activities, including anti-inflammatory (Moscatelli et al., 2006) and anticancer (Jeong et al., 2007) activities. To improve its bioavailability and oral availability, Ninomiya and his team prepared tricin-amino acid derivatives as prodrugs and investigated their cell permeability, stability *in vitro*, and oral availability *in vivo*. Among the prodrugs, compound **42** exhibited enhanced permeability with an excellent relative permeation rate of 6.3, stability in MDCK cells (*t*_1/2_ = 165 min), and increased bioavailability after oral administration with the maximum concentration of tricin being 2,497 ng/mL in the 100 mg/kg group. Additionally, icaritin exhibits various pharmacological activities, including anticancer and osteoporosis treatment (Chen et al., 2012; Zhao et al., 2015). Because this prenylflavone is very safe, it would be a good lead compound for further modification to obtain potent compounds with remarkably improved antimicrobial activities and low toxicity. Thus, Lin et al. (2017) designed a series of new hemiflavonoid-based small molecules that mimic antimicrobial peptides from icaritin to fight drug-resistant gram-positive bacterial infections. They found that compound **43** containing two arginine residues showed remarkable antibacterial activity (MICs = 1.56–3.13 μg/mL) against gram-positive bacteria MRSA. This compound has low cytotoxicity and selectivity >511, comparable to that of several antibiotics in clinical trials. Their data showed for the first time that icaritin derivatives effectively kill bacteria. Compound **43** showed rapid bactericidal activity by destroying bacterial membranes and circumventing the development of bacterial resistance (Figure 5).

**Figure 6:**
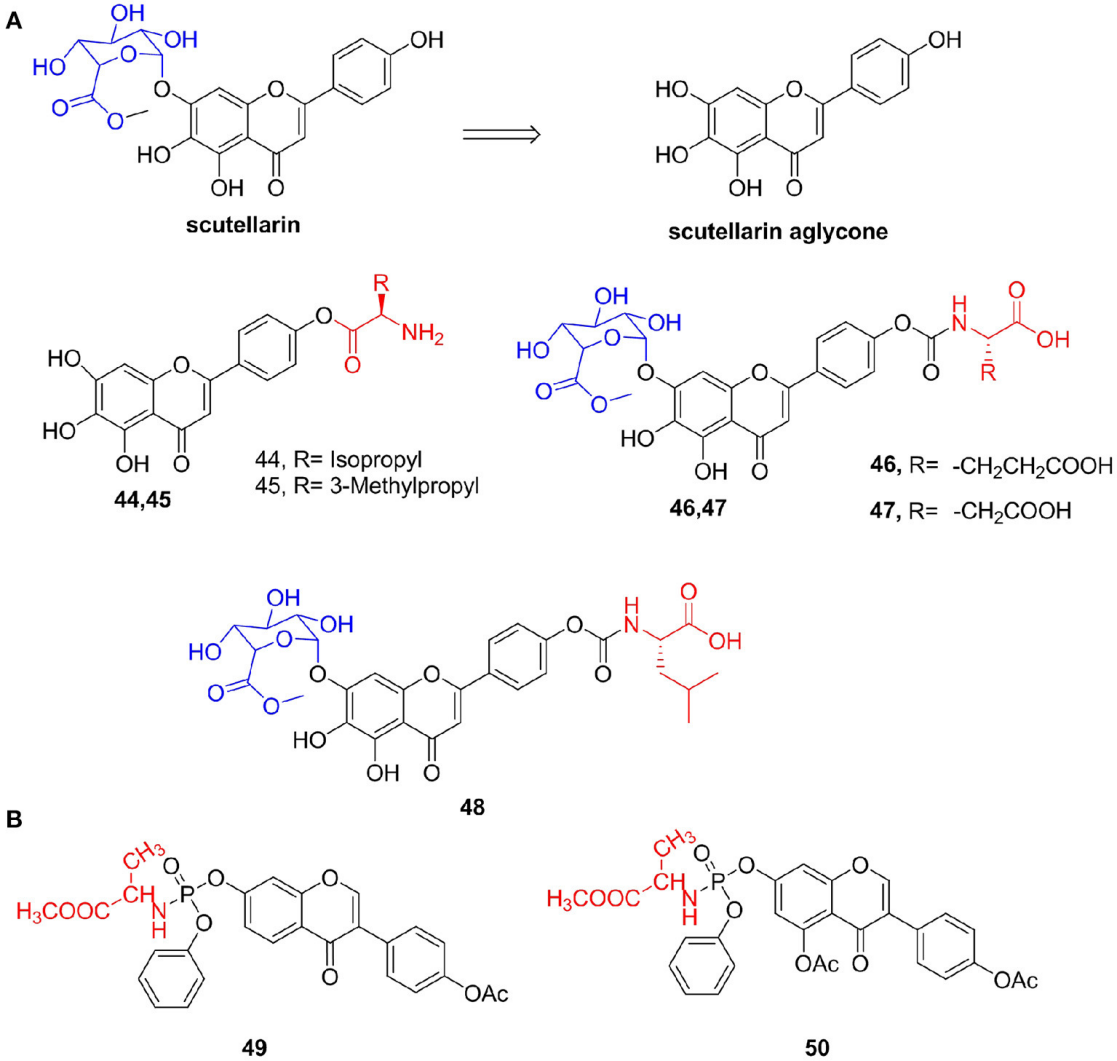
**(A)** Scutellarin amino acid derivatives. **(B)** Isoflavone amino acid derivatives.

Additionally, scutellarin is a flavonoid extracted from the Compositae plant *Erigeron breviscapus*. It is mainly used for the treatment of cardiovascular and cerebrovascular diseases (Lin et al., 2007). Results of recent pharmacodynamics and pharmacokinetic studies show that the actual form of absorption and pharmacodynamics of scutellarein in the body after oral administration is the compound itself. However, scutellarin aglycone still has defects such as low absolute bioavailability (7% in rats) and difficulty in intestinal absorption (Chen X. et al., 2006). Thus, Fu et al. (2011) synthesized *L*-amino acid ester derivatives of scutellarin aglycone and found that the water solubility levels of derivatives **44** and **45** were 1,796 and 4,100 μg/mL, respectively, which were much greater than that of scutellarin (14.4 μg/mL). Furthermore, Jiang et al. (2013) used scutellarin methyl ester and *L*-amino acid tert-butyl ester hydrochloride as raw materials to prepare a series of scutellarin methyl ester prodrugs and evaluated water solubility and the *in vitro* stability of the target compound. Their results indicated that compounds **46** (aqueous solubility = 123.1 μg/mL) and **47** (426.5 μg/mL) had better solubility and *in vitro* stability than those of scutellarin, respectively. Cha et al. (2014) also synthesized a series of 4'-*L*-amino acid derivatives of scutellarin methyl ester and reported that the designed target compounds had higher enzymatic and chemical stability and aqueous solubility. Among these compounds, compound **48** had the highest *P*_appAPtoBL_ value (1.9 × 10^−6^ cm/s) and lowest ER (*P*_appBLtoAP_/*P*_appAPtoBL_ = 0.56) and protected PC12 cells from oxidative damage induced by H_2_O_2_ by reducing the loss of MMP and reducing the generation of ROS induced by H_2_O_2_ (Figure 6A).

#### Isoflavones

Li et al. (2016) synthesized a series of isoflavone-7-phosphoramidate derivatives and determined their antiproliferative activity *in vitro* against the HepG2 cell line and L02 cell line. Compounds **49 (**IC_50_ = 5.5 μM) and **50 (**IC_50_ = 6.6 μM), which incorporated the methyl alanine, exhibited high inhibitory activities against the HepG2 cell line. Compound **49** can significantly induce G2/M arrest and lead to early apoptosis in HepG2 cells (Figure 6B).

#### Chalcones

**Figure 7:**
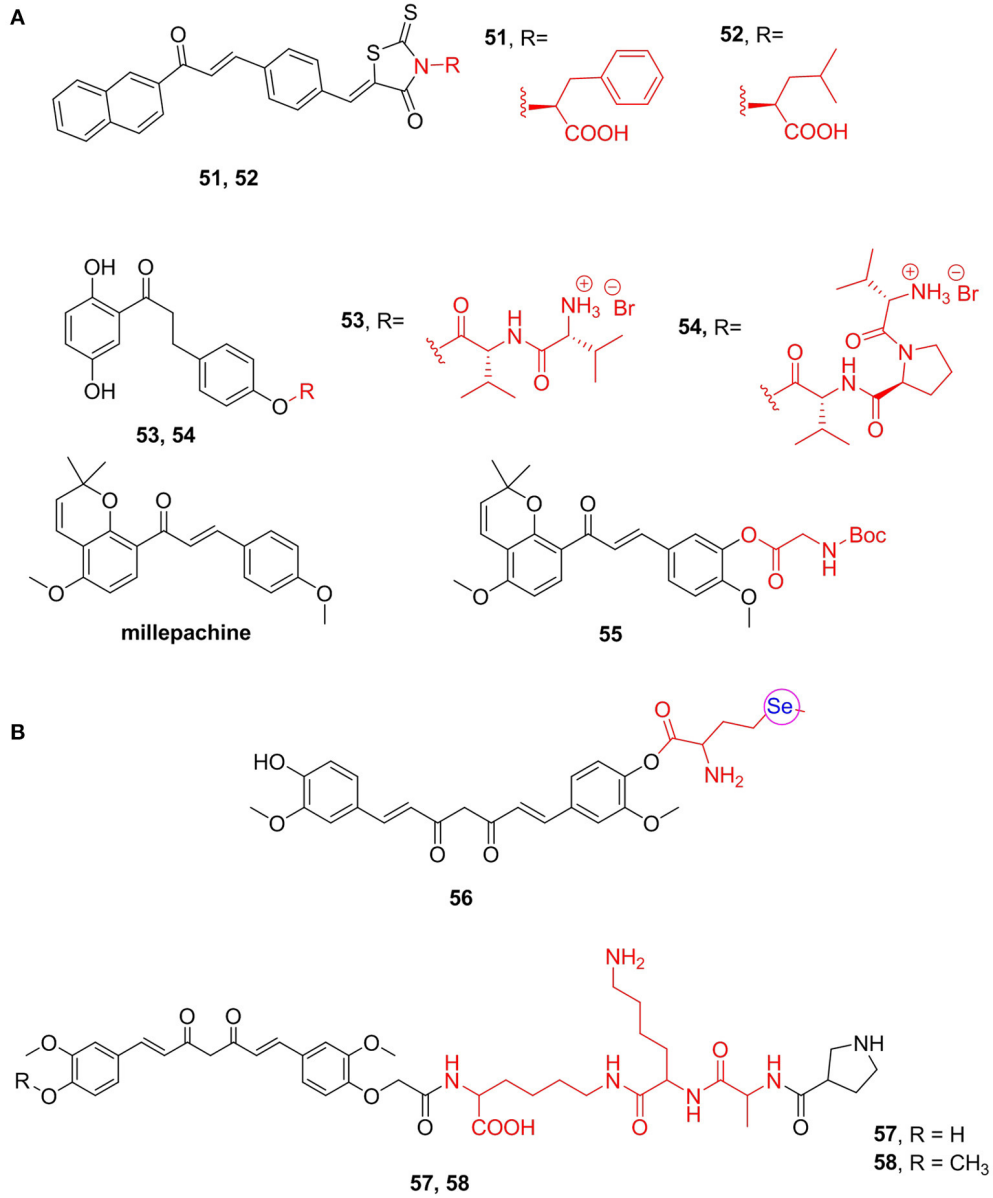
**(A)** Chalcones amino acid derivatives. **(B)** Curcumin amino acid derivatives.

Chalcones, with α,β-unsaturated ketone structures, are derived from natural compounds and chemical synthesis, possessing a variety of biological properties (Zhuang et al., 2017). Jin et al. (2012) synthesized a chalcone derivative containing *L*-phenylalanine and evaluated its antibacterial activity. Among which compound **51** (MIC = 2 mg/mL) exhibited the best antimicrobial activity against *Staphylococcus aureus* RN4220. This compound showed the most potent activity against all the MDR clinical isolates tested. Based on compound **51**, Song M. X. et al. (2014) changed the types of amino acids and synthesized another series of derivatives and found that, among all, leucine (**52**, MIC = 1 mg/mL) was the most potent against two methicillin-resistant *S. aureus* (Figure 7A).

1,3-Diphenylpropanones is a positive allosteric modulator of α7 nAChRs, which have good analgesic activity but poor pharmacokinetics. To improve its biological activity *in vivo*, Criado et al. (2016) have prepared amino acid and peptide derivatives as prodrugs of these modulators. Compounds **53** (inhibition of α7 nAChR currents = 14.7%) and **54** (inhibition of α7 nAChR currents = 24.7), prodrugs of 1,3-diphenylpropanones, showed obviously antinociceptive activity *in vivo*. Compound **54** shows roughly the same analgesic effect as the parent compound but long-lasting effects (Figure 7A).

Furthermore, millepachine is a natural pyranochalcone isolated from *Millettia pachycarpa* (Zhou Z. Y. et al., 2019). This compound has a benzopyran structure, which has good cell membrane permeability (Nicolaou et al., 2000); however, its water solubility is not optimistic. Thus, Wu et al. (2015) prepared a series of amino acid derivatives of millepachine and tested their solubility and antitumor activity. They found that compound **55**, a glycine derivative, with an IC_50_ value for MDAMB-231 cells of 0.13 μM, showed the best potency and long-term inhibition of cell viability and induced apoptosis. The solubility of compound **55** in human plasma is 2.98 mg/mL, which is significantly stronger than the lead compound millepachine (0.15 mg/mL) (Figure 7A).

Additionally, curcumin has been reported to have a variety of pharmacological activities, such as anticancer, antioxidant, and anti-inflammatory activities. However, low bioavailability, poor water solubility, and poor pharmacokinetics hinder the clinical application of curcumin (Kotha and Luthria, 2019). Thus, Cao et al. (2014) synthesized curcumin derivatives with enhanced bioactivity and reported that compounds containing one selenomethionine molecule (**56**) attached to the hydroxy group of curcuminoids showed high antimicrobial activity. Moreover, they exhibited strong DPPH scavenging ability, which was about 10 times stronger than that of curcumin and has good antiproliferative activity on a variety of cancer cells, with an IC_50_ value of <1 μM. Furthermore, Bi et al. (2016) got a new class of derivatives that can associate apoptosis induced by ischemia/reperfusion injury and reverse mitochondrial oxidative stress. The results suggest that selected curcumin polypeptide analogs **57** (EC_50/.OH_ = 25.6 μM) and **58** (EC_50/.OH_ = 31.4 μM) exhibited good hydroxyl radical scavenging activity and can facilitate the recovery of mitochondrial reticular networks and cell rescue (Figure 7B).

#### Other Flavonoids

**Figure 8:**
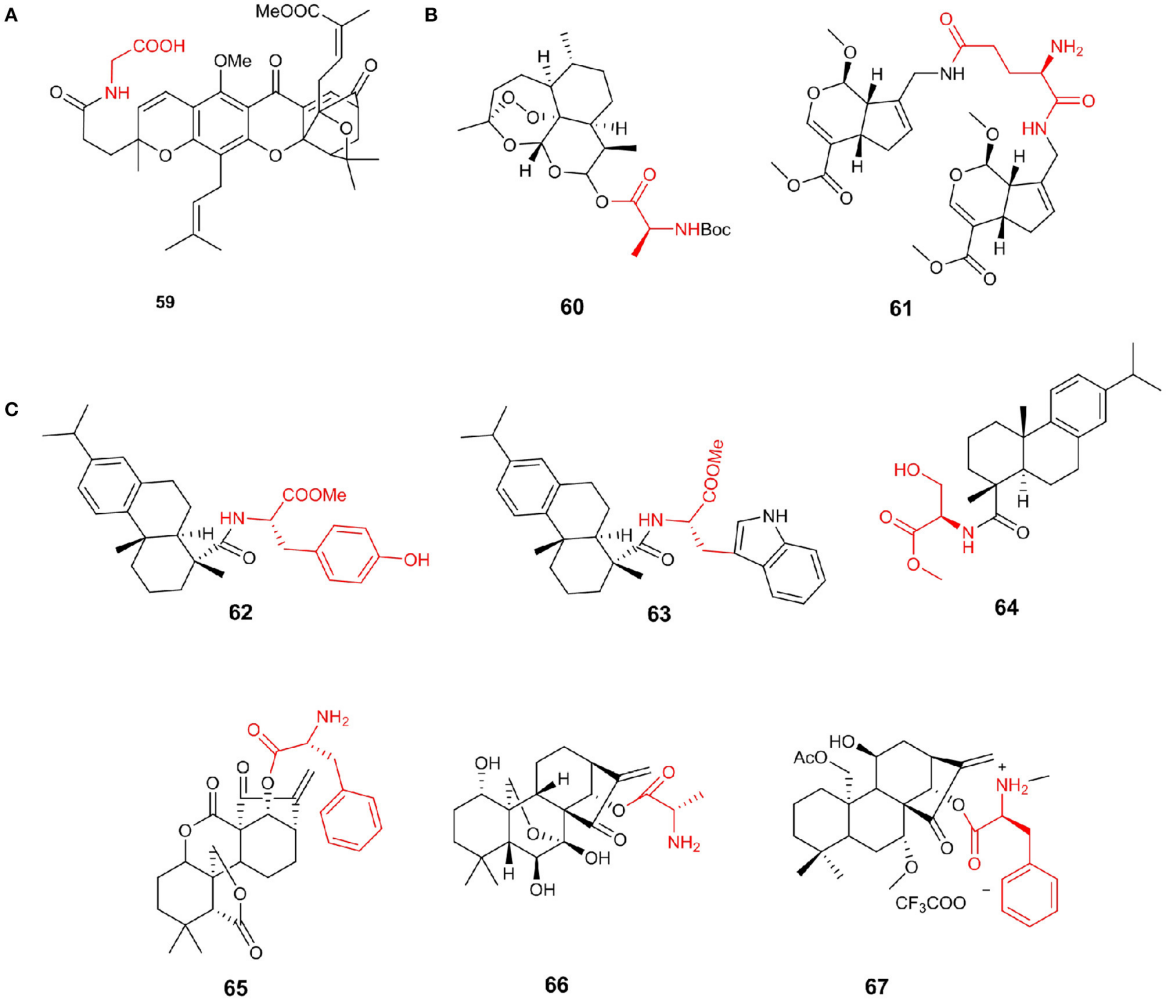
**(A)** Gambogic acid amino acid derivatives. **(B)** Sesquiterpenes amino acid derivative. **(C)** Diterpenes amino acid derivative.

Gambogic acid (GA), the most important compound of Garcinia, has been reported to be a hopeful antitumor agent. Chemical modification of GA C-37 position by introducing hydrophilic amines can increase activity (Li et al., 2012). Notably, the antiproliferative activity of compound **59** was almost 2-fold more active than that of GA against HCT 116 cell lines with an IC_50_ value of 1.35 μM (Figure 8A).

### Terpenoids

Terpenoids are the general term for polymers and their derivatives of isoprene or isopentane structural units, with general structural formula (C_5_H_8_)_n_ and a very wide range of biological activities. There are many natural products from terpenes used in clinical applications, such as the antimalarial drug artemisinin (Sowunmi et al., 2016), the antitumor drug paclitaxel (Shi et al., 2018), and the anti-infective drug andrographolide (Wen et al., 2015).

#### Sesquiterpenes

It has been reported that artemisinin has anti-Toxoplasma activity; however, the activity is not strong (Sarciron et al., 2000). Deng et al. (2020) discovered that compound **60** (selectivity index = 6.44), showed the most effective anti-*T. gondii* activity and low cytotoxicity. Furthermore, compound **60** had better growth inhibitory activity on *T. gondii* than positive control drug and remarkably reduced the number of tachyzoites *in vivo* (*P* < 0.01). The mouse Toxoplasma infection model proved the effectiveness of compound **60** anti-*T. gondii* property *in vivo* and its role in the reduction of liver damage caused by *T. gondii*. Additionally, genipin is the product of geniposide hydrolysis by β-glucosidase and has anticancer activity. Fang et al. (2018) reported genipin analogs obtained by a biomimetic synthesis strategy and evaluated their cytotoxic activity. Compound **61** was reported to have a significantly stronger inhibitory effect on tumor cell proliferation than that of cisplatin, with IC_50_ values of 0.43–1.18 μM (Figure 8B).

#### Diterpenes

Abietic and dehydroabietic acids are a class of diterpenoids that show moderate cytotoxicity. Ukiya et al. (2013) prepared amino acid conjugated derivatives from these acids to evaluate their cytotoxicities in a variety of cells. Among the derivatives, compound **62** showed potential cytotoxic activities against multiple cancer cell lines with IC_50_ values of 2.3–8.1 μM, and the cytotoxic activity of leukemia cells has the highest selectivity. It is estimated that more than 65% of all bacterial infections resistant to chemotherapy are related to biofilms (Lebeaux et al., 2013). Dehydroabietic acid was reported to prevent the formation of *S. aureus* biofilms in the low micromolar range and only 2–4-fold higher concentrations to significantly reduce the viability and biomass of existing *S. aureus* biofilms (Fallarero et al., 2013). The combination of dehydroabietic acid scaffolds and different amino acids can simultaneously target planktonic bacteria and biofilm bacteria in *S. aureus* strains and has stronger anti-biofilm agents than conventional antibiotics (Manner et al., 2015). Compound **63** was the most potent abietane-type anti-biofilm agent and exhibits strong activity on pre-formed biofilms at a concentration that is threefold higher than the concentration required to inhibit biofilm formation. Abietic acid and dehydroabietic acid, in a shortage of antibiotics and antimicrobial resistance problems looming against the growing respect, showing potential value. Helfenstein et al. (2017) discovered a dehydroabietic acid derivative **64** containing a serine side chain; its MIC_90_ against *Staphylococcus aureus* ATCC 25923 was 60 μg/mL, and the IC_50_ value on Balb/c 3T3L cells was 45 μg /mL (Figure 8C).

Oridonin was isolated from isodon with a special structural scaffold and has excellent bioactivity, including anticancer activity (Wang L. et al., 2012). However, poor solubility and moderate bioavailability of oridonin hindered its clinical research on cancer treatment. Many researchers have modified the structure of oridonin, hoping to obtain good anticancer drugs, including the candidate drug HAO472, which is undergoing phase I clinical trials. Hu et al. (2019) designed and synthesized a series of enmein-type diterpenoid amino acid ester derivatives according to HAO472 and tested their antiproliferative activity on various cancer cell lines and L-02 normal hepatocytes. The results show that compound **65** (IC_50/Bel−7402_ = 0.07 μM) has the strongest cytotoxicity against human liver cancer Bel-7402 and chronic myeloid leukemia K562 cells at the submicromolar level more potent than *L*-alanine-(14-oridonin) ester **66**(IC_50/Bel−7402_ = 9.56 μM). Simultaneously, it shows low cytotoxicity (IC_50_ for L02 cells > 50 μM) to normal cells and high selectivity. At the same time, Ke et al. synthesized a series of Flexicaulin A amino acid derivatives and evaluated their antiproliferative activity against four kinds of cancer cells. Compared with Flexicaulin A, the anticancer activity and solubility of most derivatives were remarkably improved. Compound **67** showed the most excellent activity, and its IC_50_ value for TE-1 cells was 0.75 μM. It could inhibit the proliferation of cancer cells and the formation of cell colonies and increase the level of ROS in TE1 cells (Figure 8C).

#### Triterpenoids

*Panax ginseng* is widely used as a healthy functional food due to its obvious health benefits. Ginsenosides are the main bioactive constituents of *Panax ginseng*. Panaxadiol (PD), protopanaxadiol (PPD), 25-methoxyprotopanaxadiol (AD1), and 25-hydroxyprotopanaxadiol (AD2) are obtained by hydrolysis of ginsenosides and have a variety of biological activities, including anticancer (Zhang et al., 2007; Salvador et al., 2017), antiliver fibrosis (Han et al., 2018), and antibacterial (Amoussa et al., 2016) activities. Many researchers have introduced amino acids into ginsenosides and obtained ginsenoside amino acid derivatives with enhanced activity. Liu et al. (2011) synthesized a series of PD amino acid derivatives by modifying the 3-hydroxyl group of panaxadiol and evaluated their activities against a panel of human tumor cell lines. All amino acid derivatives showed stronger antiproliferative activities than PD on the growth of different cancer cells *in vitro*. From the results of the antitumor tests in HepG2, compound **68** (IC_50_, 5.03 μM) exhibited 7.5 times the strongest activities than that of PD (IC_50_, 37.91 μM). For the A549 cell line, compound **69** (IC_50_, 7.63 μM) showed the strongest activity, which was 5.6 times stronger than PD (43.13 μM). Ocotillol enantiopure and its derivatives showed good antibacterial activity anti-gram-positive bacteria (Bi et al., 2015; Ren et al., 2019), and they can be obtained by the oxidation of PPD with *m*-CPBA. Wang et al. (2018) introduced free amino groups at the C-3 position to confirm whether the group can enhance the antibacterial activity. The results showed that compound **70** showed excellent antibacterial activity with MIC of 2 μg/mL against MRSA USA300 and 4 μg/mL against *B. subtilis*. Derivatives of AD2 were measured on six distinct human tumor cell lines (Wang et al., 2013). Compound **71** exhibited the best antitumor activity with IC_50_ values ranging from 2.77 to 6.83 μM for six cell lines compared to the parent AD2. Qu et al. (2015) continued to synthesize AD-2 amino acid derivatives and evaluated the *in vitro* antitumor activities of five human tumor cell lines. Compounds **72** (IC_50_ = 4.2–9.9 μM) and **73** (IC_50_ = 4.2–7.1 μM) displayed the most potent anticancer activities. The two derivatives could remarkably induce the apoptosis of human prostatic carcinoma DU145 cells and have low toxicity in IOSE144 cells and HOSEpiC cells. Yuan et al. (2018) modified AD1 and AD2 with non-protein amino acids and obtained two series of derivatives of AD1 and AD2. Compounds **74** and **75** exhibited strong biological activity against HCT-116 and BGC-823 cell lines, with_IC_50_ values in the range of 4.76–9.03 μM, and exhibited higher cytotoxic activity than AD-2 and AD-1. The relative plasma stability of esters is closely related to the hydrolytic enzymes in plasma. It is reported that the increase in steric hindrance near the hydrolyzable group will reduce the affinity between the compound and the hydrolase, thereby improving the plasma stability of the compound (Borthwick et al., 2003; Carroux et al., 2013). Therefore, among these ginsenoside compounds, the plasma stability of compound **70** should be the strongest.

**Figure 9:**
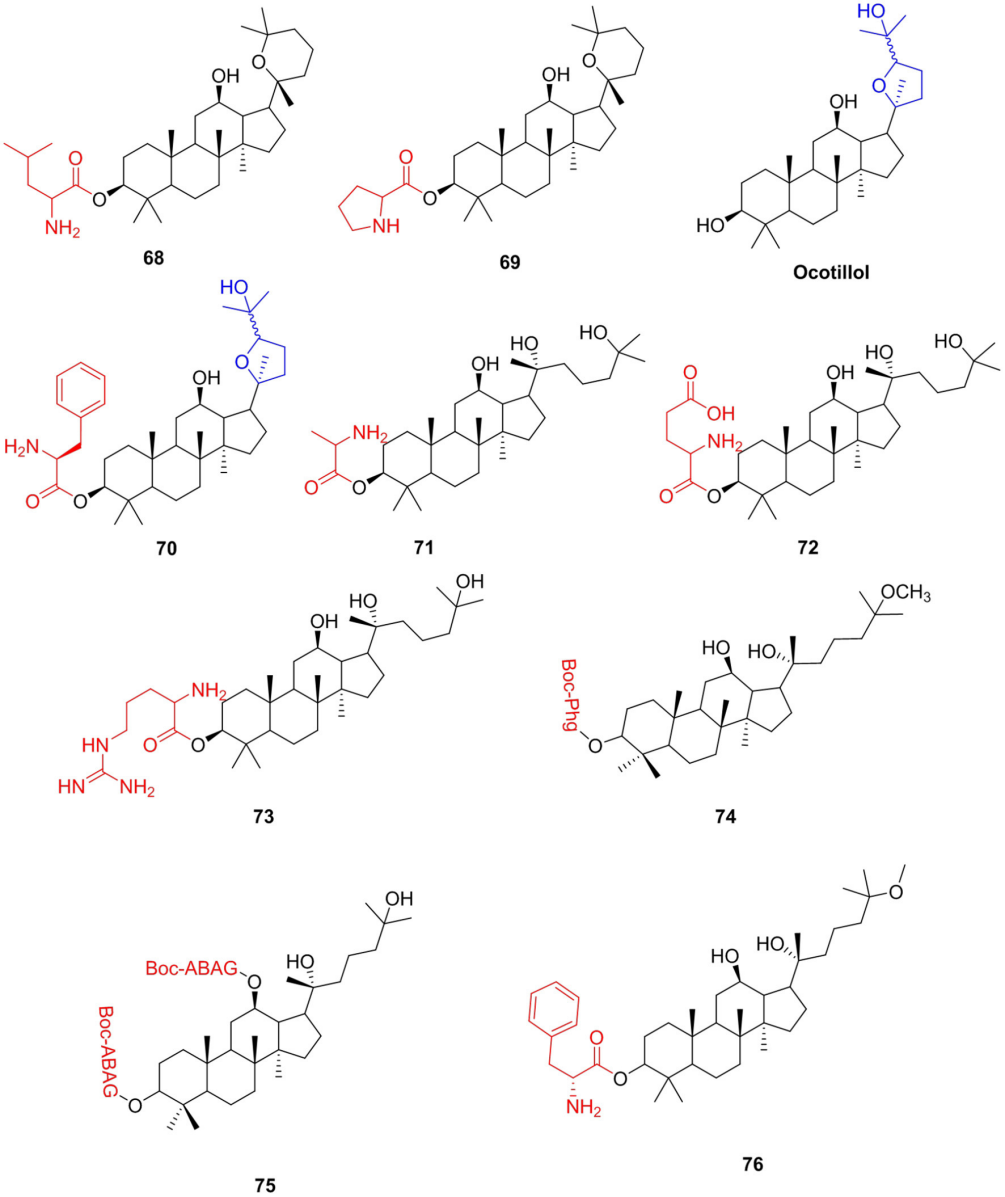
Ginsenosides amino acid derivatives.

Research has demonstrated that AD-1 could regulate c-FLIP pathway-mediated activation of NF-κB, thereby showing liver protection and reversal of activated HSC (Wu et al., 2011). At the same time, AD-1 improved hepatic injury, fibrosis, and inflammation induced by thioacetamide in C57BL/6 mice (Han et al., 2018). The present study aimed to introduce amino acids to AD-1 and obtained safer, more effective, and developable derivatives, which could be used to prevent or improve liver fibrosis, and simultaneously provide amino acid dietary supplements. Conjugate **76** has a significant inhibitory effect on cell proliferation in activated t-HSC/Cl-6 cells (IC_50_ = 3.8 μM) and appeared to be non-toxic to L02 cells (Figure 9).

23-Hydroxybetulinic acid existed in the root of *Pulsatilla chinensis*, which has anti-HIV and antitumor activities similar to betulinic acid and botulin (Bi et al., 2007; Zhu et al., 2009). Unfortunately, both betulinic acid and botulin are very poorly soluble in aqueous buffers; thus, their bioavailability and biodistribution are inadequate in terms of medical applications (Drag-Zalesinska et al., 2009). Suitable modification at the right positions of natural products may yield analogs with significantly improved potency. A set of 17-carboxylic acid-modified 23-hydroxybetulinic acid derivatives that displayed slightly improved antiproliferative potencies were reported previously (Bi et al., 2007). To increase the anticancer activity and structural diversity of 23-hydroxybetulinic acid, Lan et al. (2011) prepared a series of new compounds and screened these compounds for *in vitro* antiproliferative activity against various cancer cell lines. The amino acid derivatives **77** and **78** with IC_50_ <10 μM in all tested cell lines were considered to be the most hopeful compounds.

**Figure 10:**
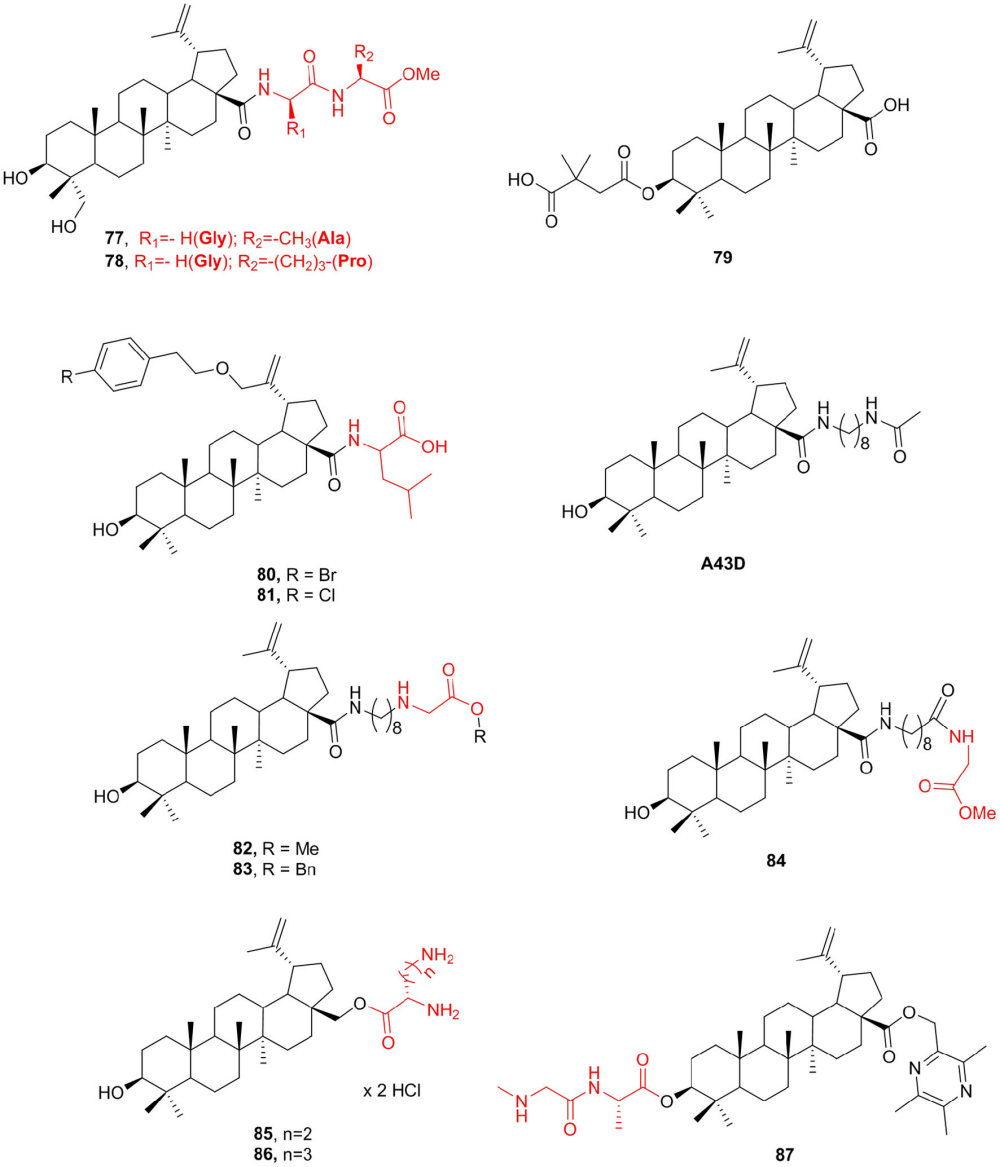
Betulinic acid derivatives.

Betulinic acid exhibits anticancer and anti-HIV biological activities, and its derivative Bevirimat (**79**) is undergoing phase II clinical trials against HIV (Smith et al., 2007). Bevirimat exhibited promising pharmacokinetic profiles in clinical trials, but its effectuality was compromised by the high baseline resistance of HIV-1 variants with polymorphism. A series of betulinic acid (BA) derivatives were measured for their inhibition of the chymotrypsin-like activity of the 20S proteasome (Qian et al., 2011). Compounds **80** and **81**, comprising leucine, illustrated the best proteasome inhibition activity with IC_50_ values of 1.56 and 1.80 μM, respectively, which are 3–4-fold more potent than the proteasome inhibition controls LLM-F and lactacystin. To determine whether the viruses with bevirimat-resistant polymorphism also altered their sensitivity to betulinic acid derivatives that inhibit HIV-1 entry, a series of new betulinic acid inhibitors were tested for their activities against HIV-1 NL4-3 and NL4-3 variants resistant to bevirimat (Dang et al., 2012). The results show that the bevirimat-resistant viruses were ~5–10-fold more sensitive to three new glycine ester derivatives **82**, **83**, **and 84**, and showed potent anti-HIV activity with EC_50_ ranging from 0.04 to 0.12 μM. In contrast, wild-type NL4-3 and bevirimat-resistant variants were equally sensitive to the HIV-1 RT inhibitor AZT. Compound A43D exhibited the most potent anti-HIV-1 entry activity (Huang et al., 2006). Compared with A43D (CL_int_ = 0.37 ml/min/mg), these three new compounds **82** (CL_int_ = 0.23 ml/min/mg), **83** (CL_int_ = 0.17 ml/min/mg), and **84** (CL_int_ = 0.22 ml/min/mg) showed significantly improved microsomal stability (Figure 10).

The new amino acid derivatives of betulinic acid and betulin were synthesized, and their anticancer activity was evaluated (Drag-Zalesinska et al., 2015). In particular, compounds containing a lysine side chain (**85**, IC_50_ = 7 μM) and ornithine (**86**, IC_50_ = 10 μM) are stronger than the parent compounds, and the biggest advantage was that new compounds are safe for normal human keratinocytes. In addition, the literature reports that the introduction of amino acids into triterpenes could enhance selective cytotoxicity and water solubility (Schwarz et al., 2014). Based on the above, researchers attempted the synthesis of several ligustrazine-betulinic acid (TBA) amino acid derivatives BAX by introducing several amino acids to the C3 of TBA to enhance its antitumor activities and tumor targeting. These compounds were screened for selective cytotoxic activity against five cancer cell lines and the non-malignant MDCK cell line. Most of the tested TBA-amino acid derivatives showed better antiproliferative activity against all tumor cell lines than TBA. Among them, compound **87** showed the best cytotoxic activity on tumor cell lines (IC_50_ = 2.31 μM), which was 2-fold higher than that of the positive drug cisplatin (DDP), while it showed lower cytotoxicity in MDCK cells than DDP cells (Figure 10).

**Figure 11:**
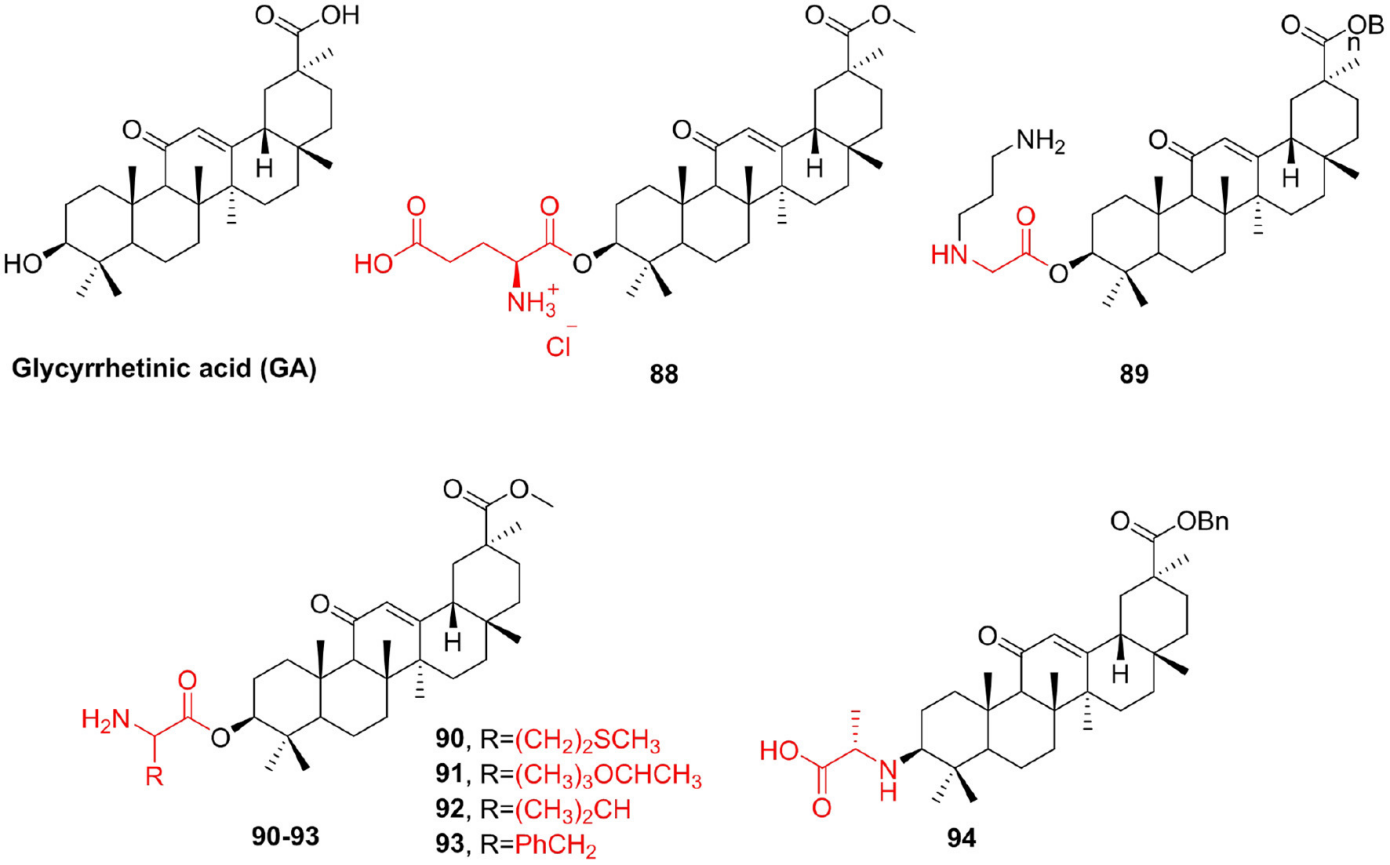
Glycyrrhetinic acid derivatives.

Glycyrrhetinic acid (GA) is the main component of licorice root (Baltina, 2003; Schwarz and Csuk, 2010); its antitumor activity is low compared with other members of the triterpenoid family (Tatsuzaki et al., 2007). GA is easily inexpensive and shows apoptotic effects on tumor cells. These facts make GA and its derivatives the focus of our scientific interest. In addition, GA demonstrated only poor selectivity (Schwarz et al., 2010). Here, we tried to enhance the poor cytotoxicity of GA via simple derivatization. Csuk et al. (2011a) make use of various glutamyl and aspartyl substituents for the synthesis of C esters of GA methyl ester. Compound **88** (IC_50_ = 1.27–2.33 μM) demonstrated 67-fold higher cytotoxicity and an up to 140-fold better selectivity toward tumor cells than that of GA. To improve the cytotoxicity of GA, Csuk et al. (2011b) synthesized new derivatives of GA, differing in structure and lipophilicity. The most active compound **89**, a benzyl glycyrrhizinate with an extra 3-N-(3aminopropyl)glycyl substituent, showed an IC_50_ between 1.96 and 5.14 μM for five human cancer cell lines and triggers apoptosis in 80% of the cells. Use various amino acids, including methionine (**90**), threonine (**91**), valine (**92**), and phenylalanine (**93**), to modify glycyrrhetinic acid to obtain more effective derivatives (Wang J. et al., 2012). These compounds could protect the growth of *E. coli, Bacillus subtilis*, and yeast against high concentrations of DMF. To improve the cytotoxicity of GA and explore the effect of the bonding mode on antitumor activity, Zhou F. et al. (2019) synthesized a series of amino acid derivatives and evaluated their antitumor activity. All the deprotected (without Boc group) derivatives displayed much stronger cytotoxic activity than GA, and surprisingly enough, all the GA-NH2 series of the compounds were more effective than GA-OH series against different tumor cells. Among them, compound **94** showed better antitumor activity against A549 (IC_50_ = 2 μM) than cisplatin (IC_50_ = 9 μM). Further studies indicated that compound **94** could induce A549 apoptosis by nuclei fragmentation and prevented the transition from S to G2 phase (Figure 11).

**Figure 12:**
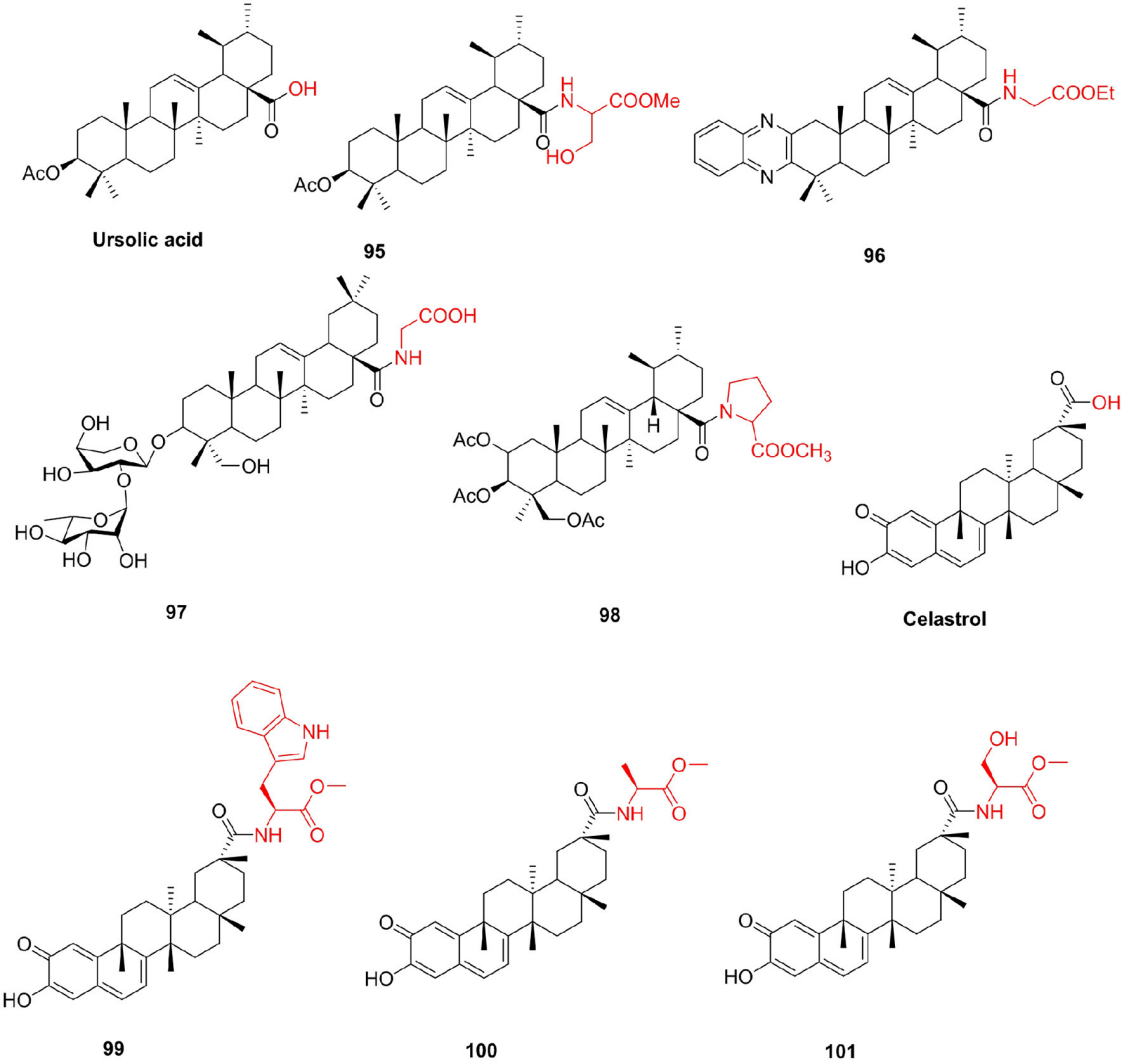
Other triterpenoids derivatives.

In addition to those listed above, other pentacyclic triterpene compounds often react with amino acids to improve their unfavorable properties. Ursolic acid is abundant in the plant kingdom. Ursolic acid and its derivatives have been reported to have interesting bioactivities (Choi et al., 2000; Chattopadhyay et al., 2002). Shao et al. (2011) synthesized some ursolic acid derivatives and determined their cytotoxic activity on various cancer cells. Among them, the derivative **95** introduced into serine showed greater anti-A549 cell activity than ursolic acid, with an IC_50_ of 35.3 μM. Fu H. J. et al. (2014) prepared a series of new ursolic acid derivatives under the guidance of virtual screening and used RBL2H3 cells and ovariectomized (OVX) rats for biological evaluation. Compound **96**, which contains a glycine structure, showed potent inhibitory activity on serotonin biosynthesis, which inhibited the protein and mRNA expression of Tph-1 and lowered serotonin contents in the serum and gut without influencing brain serotonin. Due to their strong hemolytic activity, the clinical development of Pulsatilla saponins A (PSA) and D (PSD) is limited (HD_50_ = 10 μM) (Wang and Fang, 2009). To solve the problem, Chen et al. (2015) prepared C-28 position derivatives of PSA/PSD and evaluated the hemolytic activity and cytotoxicity of these compounds. Compared with PSA, the acute toxicity of compound **97** to mice is greatly reduced. At the same time, compound **97** (HD_50_ > 500 μM) is a potential antitumor agent, thereby preventing hemolysis (Figure 12).

Asiatic acid, an active pentacyclic triterpenoid found in *Centella asiatica*, can be easily prepared by the hydrolysis of asiaticoside. Asiatic acid also has many biological effects (Mook-Jung et al., 1999; Park et al., 2005), but the efficacy of the original asiatic acid is relatively poor. Jing et al. (2015) synthesized the derivatives of Asiatic acid and evaluated their biological activities. The derivative **98**, containing the proline structure, had markedly better antitumor activity (IC_50_ < 10 μM) than both Asiatic acid and other derivatives, with stability similar to that of its parent compound Asiatic acid. Celastrol has many important pharmacological activities, including anticancer (Zheng et al., 2014), anti-inflammatory (Jia et al., 2015), and resistance to neurodegenerative disease activity (Paris et al., 2010), but it has several drawbacks such as poor water solubility, high toxicity, and poor stability, which restrict its clinical application. Zhang et al. (2017) designed and synthesized new celastrol derivatives and evaluated their anticancer activities. In the series of amino acid derivatives, compound **99** showed the highest anticancer activity against AGS cell lines (IC_50_ = 0.44 μM) and showed better anticancer activity against HCT-116, BEL-7402, and HepG-2 cell lines than the other compounds. Eight novel celastrol amino acid derivatives were synthesized, which displayed strong cytotoxic activities against A549, HeLa, and HepG-2 cell lines (Pang et al., 2018). In particular, the cytotoxicities of **100** and **101** against HeLa and A549 cells with IC_50_ values ranging from 0.109 to 0.371 μM, the effect of cytotoxic activity was remarkably stronger than that of celastrol and cisplatin. The most potent compound **101** showed 10- and 55-fold better antiproliferative activity of anti-A549 cell than celastrol and cisplatin, respectively. Compounds 100 and 101 can induce apoptosis in A549 cells at the concentration of 0.2 μM (Figure 12).

### Alkaloids

**Figure 13:**
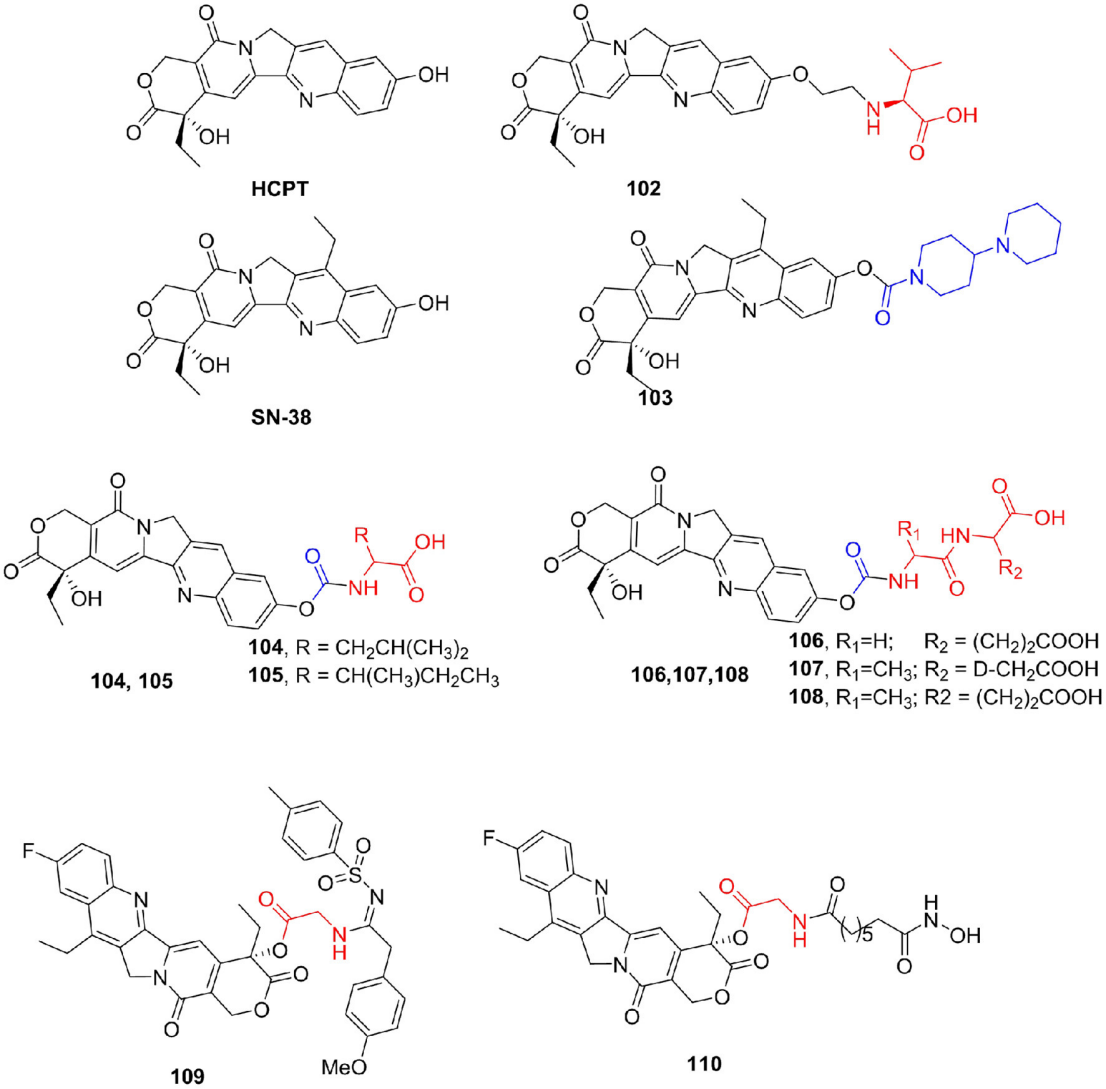
Camptothecin derivatives.

Alkaloids are a class of nitrogen-containing natural compounds with physiological effects. Most of them have complex nitrogen heterocyclic structures, and a few are non-nitrogen heterocyclic organic amines. Alkaloids are widely distributed in nature, especially in the plant kingdom, but also in the animal kingdom, such as bufotenine from toads (Kostakis and Byard, 2009) and hypoxanthine from earthworms (Huang et al., 2016). Camptothecin (CPT) is a plant anticancer drug extracted from *Camptotheca acuminata* (Pu et al., 2020), which plays an important role in clinical cancer treatment, and its derivatives are a favorite of pharmaceutical chemists. CPT mainly forms a ternary complex by interacting with DNA and topoisomerase I, blocking DNA replication, leading to cell death, and thus showing significant antitumor activity (Fan et al., 1998; Cincinelli et al., 2013). The shortcomings of CPT, such as poor solubility and large side effects, have seriously affected its clinical application (Ye et al., 2012). Many researchers have synthesized a large number of CPT derivatives, hoping to find targets with fewer side effects. 10-Hydoxycamptothecin (HCPT) is a natural CPT derivative that was approved for marketing in 1988 (Guerrant et al., 2013). Meng et al. (2014) synthesized a series of amino acid derivatives to improve the antitumor activity and reduce the side effects of irinotecan. Among them, the derivative **102** (IC_50_ for KB cell line = 1.39 nM, LogP = 0.74) of valine and HCPT not only maintained the tumor proliferation inhibitory activity equivalent to HCPT (IC_50_ for KB cell line = 1.48 nM), but also its water solubility was significantly improved compared with LogP of HCPT of 1.53 (Figure 13).

Irinotecan (**103**) is a water-soluble prodrug of the CPT derivative SN-38. However, high individual variation in efficacy, toxicity, and side effects of irinotecan preclude its clinical use (Dodds and Rivory, 1999). Novel derivatives of SN-38 were synthesized by conjugating amino acids to the 10-hydroxyl group via a carbamate linkage (Zhou et al., 2014). All prodrug compounds showed much greater *in vitro* antitumor activities against SGC-7901 cells and HeLa cells than irinotecan. The most active compounds **104–107** exhibited IC_50_ values that were 1,000 times lower against HeLa cells and 30 times lower against SGC-7901 cells than that of irinotecan, and the inhibitory activities of these prodrugs against AchE were remarkably reduced, with IC_50_ values more than 6.8 times better than that of irinotecan. In addition, compound **108** showed the same level of tumor growth inhibitory activity (Inhibitory Rate = 51%) as irinotecan (Inhibitory Rate = 51%) *in vivo*. In an attempt to improve the antitumor activity of camptothecins (CPTs), novel 10-fluoro-CPT derivatives were designed, synthesized, and evaluated for cytotoxicity against various human cancer cell lines (Yang et al., 2017). Remarkably, compound **109** (IC_50_ = 99.2 nM) displayed the strongest cytotoxicity against the MDR KB-VIN cell line. Yu et al. (2012) prepared a series of CPT prodrugs that exhibit histone deacetylase (HDAC) inhibition activity based on the synergistic effect between camptothecin derivatives and HDAC inhibitors. Compound **110** exhibited strong antiproliferative activity in A549 (IC_50_ = 137.7 nM) and HCT-116 (IC_50_ = 30.5 nM) cell lines (Figure 13).

Based on the reported anti-Leishmanial activity of the natural alkaloid piperine (Kapil, 1993), the combination of piperine is synthesized by hydrolyzing piperine to piperonic acid and then reacting with amino acid methyl ester (Singh et al., 2010). All the compounds showed better activity than piperine. Piperoyl-valine methyl ester (**111**) was the strongest active compound with an IC_50_ of 0.075 mM against the amastigotes.

**Figure 14:**
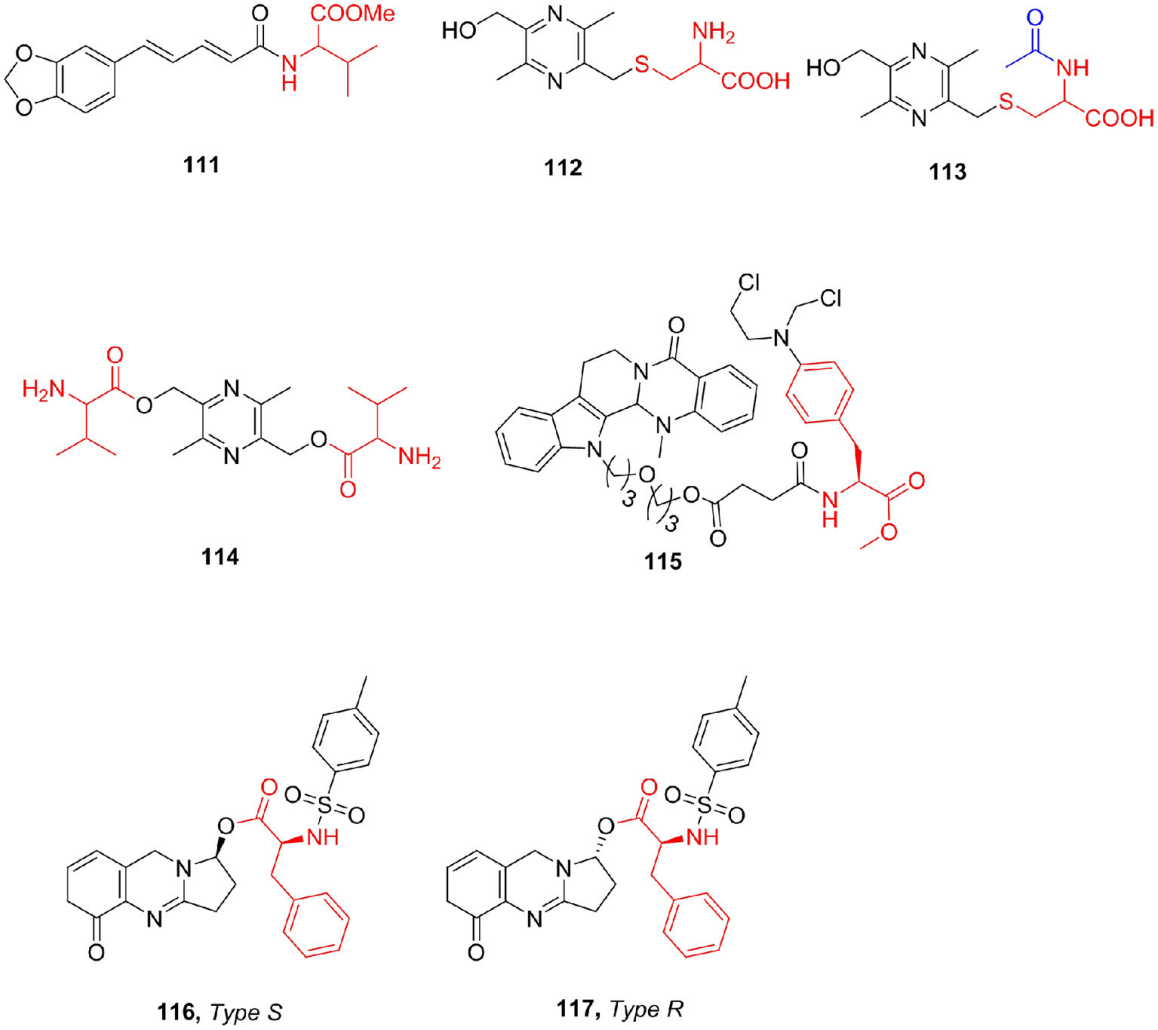
Alkaloids amino acid derivatives.

Liguzinediol (Ligu) is a para-xylene derivative of ligustrazine, an alkaloid compound, which is a potential treatment for heart failure, but its safety is low (Liu et al., 2009). To determine whether Ligu metabolites have positive inotropic effects, Zhu et al. (2018) synthesized two amino acid-linked metabolite derivatives (**112, 113**). Through pharmacological evaluation, Ligu's positive inotropic activity is mainly since the parent drug and its metabolites have no adverse reactions such as arrhythmia. Through pharmacokinetics, it was found that Ligu is eliminated faster *in vivo* and has a shorter half-life. After studying the structure–activity relationship between Ligu metabolites *in vivo*, Li et al. (2017) synthesized Ligu valine ester (**114**) prodrugs and determined the solubility of the compound (224 mg/mL) and the ester–water partition coefficient (−0.47). The experimental results show that the amino acid ester prodrug retains the solubility characteristics of the original drug, and the *in vivo* and *in vitro* experiments have obtained good experimental results, which significantly prolong the *in vivo* action time of Ligu; but the fat solubility of the compound was not improved (Figure 14).

Evodiamine is an indole alkaloid that exists in the fruits of the traditional Chinese herb *Evodiae fructus*, which is widely used to treat a variety of human diseases (Yu et al., 2013). Although evodiamine showed good antitumor activity, the development of evodiamine for clinical cancer treatment was stunted by its relatively moderate potency. Hu et al. (2017) reported a series of novel evodiamine with antiproliferative properties. All derivatives showed antiproliferative activities against tested human tumor cell to some extent and almost non-toxic (>200 μM) to human normal PBMCs. Compound **115** showed the strongest cytotoxicity against two tumor cell lines (THP-1 and HL-60) with IC_50_ values of 4.05 and 0.50 μM, respectively, and selected for further mechanism study in HL-60 cells, and compound **115** could induce HL-60 cell apoptosis and arrest at the G2 phase at low concentrations. (±)-Vasicinone was isolated from the seeds of *P. harmala*. Filali et al. (2019) reported the synthesis of a new series of α-amino acid ester derivatives of (±)-vasicinone and tested for their *in vitro* AChE, anti-5-LOX, and cytotoxic activities (MCF-7, OVCAR-3, and HCT-116 cell lines). Most of the derivatives showed cytotoxic activity against the three cell lines used. Only two diastereoisomers, **116 and 117**, exhibited activity against the 5- LOX enzyme with IC_50_ values of 63 and 79 μM, respectively (Figure 14).

### Polyphenols

**Figure 15:**
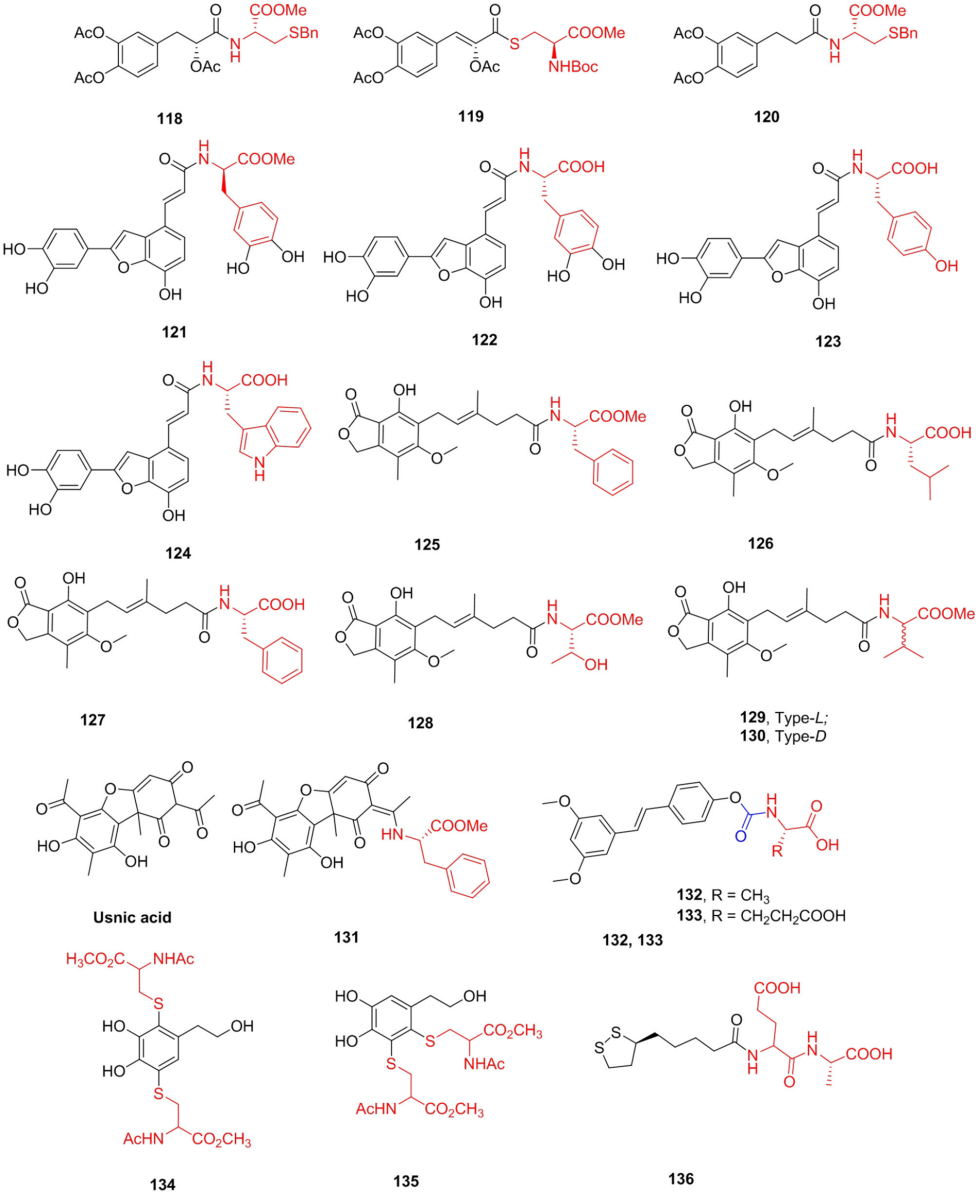
Polyphenols amino acid derivatives.

Danshensu is one of the main water-soluble active ingredients of *Salvia miltiorrhiza* Bunge (Danshen), which has been applied in clinical practice for centuries in Asia (Zhang et al., 2010). After the hydroxyl group of Danshensu is esterified or etherified, Danshensu is not easily oxidized, fat solubility is improved, and toxicity is reduced. Jia et al. (2012) designed and synthesized a series of novel amide and thioester conjugates between Danshensu and cysteine derivatives under the guidance of “medicinal chemical hybridization” (MCH). The amide conjugates **118–120** displayed widely protective effects against H_2_O_2_-HUVECs. Pretreatment with these derivatives could increase GSH levels and decrease MDA levels. Salvianolic acid C, a polyphenolic benzofuran derivative that exists in Danshen, is reported to have a potent XO inhibitory activity (Fu Y. et al., 2014). Nevertheless, few have attempted to study salvianolic acid C except for the total synthesis and pharmacokinetic study of rat plasma (Shen et al., 2012). A series of 2-arylbenzo[b]furan derivatives were prepared based on salvianolic acid C and tested for XO inhibitory and antioxidant activities (Tang et al., 2016). Compounds **121–124** showed effective XO inhibitory activities with IC_50_ values ranging from 4.0 to 6.4 mM, which were comparable with that of allopurinol. The representative derivative **123** could bind to either XO or the xanthine oxidase–xanthine complex, showing a mixed-type competitive mechanism (Figure 15).

Mycophenolic acid (MPA), first isolated in 1896 by Gosio from *Pennicillium stoloniferum* and was probably the first antibiotic (Bentley, 2000), is one of the most effective inhibitors of *h*IMPDH (human IMPDH; *K*i = 7 nM) (Hedstrom, 2009). MPA reduces the availability of guanine nucleotides, especially GTP. Mycophenolate mofetil (MMF, CellCept) and mycophenolate sodium (MPS, Myfortic) are used clinically as immunosuppressants. Many structural modifications were made to MPA, but only a few showed similar or better immunosuppressive activity. Iwaszkiewicz-Grzes et al. (2013) synthesized 11 amino acid derivatives of MPA and evaluated new analogs as growth inhibitors of Jurkat cells and *h*PBMCs cells from healthy donors. The cytotoxicity depends on the substituent and configuration of the chiral center in the amino acid unit. Compounds **125** (SI = 2871.4)**, 126** (SI = 33,787.9), and **127** (SI = 7166.7) exhibited higher potency than MPA *in vitro*. In addition, Siebert et al. (2018) obtained novel amino acid MPA derivatives as potential antibacterial agents. Peptide derivatives proved to be the most versatile, and their strains were relative to most MIC values lower than MPA. Among them, the MIC value of compound **128** against *S. aureus* MSSA ATCC 25923 was 8 μg/mL, which was almost equivalent to kanamycin (MIC = 7.8 μg/mL) and significantly stronger than that of MPA (MIC > 178 μg/mL). As can be seen, the activity of amino acid derivatives depends on the configuration at the chiral center in the amino acid unit, and methyl esters revealed better antimicrobial activity than derivatives with free carboxylic groups. Felczak et al. (2014) prepared mycophenolic adenine dinucleotide analogs linked by amino acids. Mycophenolic-(*L*)- and (*D*)-valine adenine diamide derivatives **129** (*K*i = 9 nM) and **130** (*K*i = 3 nM) were found to be very potent enzymatically but did not inhibit the proliferation of cancer cells (Figure 15).

Usnic acid (UA), isolated from lichen, has a variety of biological activities (Galanty et al., 2017), including antiparasitic activity. However, the toxicity of usnic acid, especially liver failure (Piska et al., 2018) and cultured mouse hepatocyte necrosis (Foti et al., 2008), has also been reported. Nevertheless, poor solubility in water (Erba et al., 1998) limits the application of usnic acid to some extent. Guo et al. (2019) synthesized some (+)-usnic acid derivatives containing amino acids and evaluated their anti-*Toxoplasma* activity. Compared with positive control drugs sulfadiazine (selectivity 1.15), pyrimethamine (selectivity index = 0.89), spiramycin (selectivity index = 0.72), and the lead compound (+)-usnic acid (selectivity index = 0.96), compound **131** showed the most effective anti-*T. gondii* activity (selectivity index > 2.77). Furthermore, compound **131** had better inhibitory effects on *T. gondii* (inhibition rates 76.0%) *in vivo* than spiramycin (inhibition rate 55.2%). The number of tachyzoites was significantly reduced (*p* < 0.001) and reduced hepatotoxicity and significantly increased antioxidative effects in comparison to the normal group (Figure 15).

The peroxisome proliferator-activated receptor subtype α (PPARα) has been established as a target in drug discovery studies of new lipid-lowering drugs. Pterostilbene is a naturally occurring PPARα agonist that has been shown to decrease plasma lipid concentrations through activation of PPARα (Kim and Chong, 2013). Of the conjugates investigated, 4-OMe-pterostilbene had a lower activating effect than pterostilbene, but the pterostilbenes with either amino acids **132** (Fl = 18.4) and **133** (Fl = 18.9) demonstrated a small but remarkable increase in PPARα activity compared with pterostilbene (Fl = 17.1) (Figure 15).

Polyphenols are one of the biologically active substances in many plant-derived foods. They have effective reducing properties, which can inhibit the oxidative deterioration of food. Previous attempts have been made to use this polyphenol to avoid oxidative browning of MbO_2_. However, the effective antioxidant polyphenols accelerated the oxidation of MbO_2_ to MetMb (Masuda et al., 2013). Honda et al. (2016) synthesized some polyphenols linked to amino acid compounds. Dydroxytyrosol substituted with dicysteine **134** and **135** showed the most effective reduction effect on MetMb and no oxidation effect on MbO_2_ (Figure 15).

Lipoic acid (LA) is a pharmacophore with unique antioxidant (Whiteman et al., 1996) and cytoprotective properties (Pugazhenthi et al., 2007). Based on its chemical and biological properties, LA is an attractive candidate for modification. Kates et al. (2014) prepared and screened a selection of LA analogs for cytoprotective activity. Compound **136** was selected based on these attributes as well as a favorable ADMET and receptor screen profile for further preclinical and clinical investigation, which showed statistically remarkable protection against myocardial damage associated with percutaneous coronary intervention (Figure 15).

## Conclusion

Natural products have long been considered an important source of effective therapeutic drugs. About 60% of the world's population relies on herbal medicines and natural medicines to treat diseases. With natural products as lead compounds, the relationship between structure and biological activity and metabolic research can be further carried out for structural modification and analog synthesis, which can improve drug efficacy and reduce toxicity. Amino acids are an important class of bioorganic molecules. They have the characteristics of structural diversity, ease of availability, and easy connection with other amino acids and biologically active molecules. They can effectively change the low solubility and biological activity of natural products. The introduction of amino acids into natural compounds can often improve the solubility and increase the pharmacological activities of natural products, and a few amino acids are used to increase the selectivity of these products. Certain compounds, such as anthraquinones and flavonoids, are difficult to dissolve in water. The introduction of amino acids into their structures and the preparation of amino acid hydrochlorides can greatly improve their solubility. Some compounds have strong activity. For example, PPT lacks favorable selectivity to normal cells, which leads to its increased toxicity and side effects. After the introduction of amino acids, its selectivity increases and cytotoxicity to normal cells decreases. Regarding the amino acid-protecting group, *N*-Boc, there is no clear conclusion about whether it needs to be deprotected at the end when the structure is modified.

According to previous studies, natural products have diverse structure types and pharmacological activities. For compounds with weak biological activity and low water solubility, we can first consider amino acids. For compounds with strong activity and high toxicity, we can consider adding amino acids to prepared prodrugs to reduce toxicity. When we modify the structure of a natural compound, we should first fully understand the various properties of the natural product, such as solubility, reported pharmacological activity, and structural modification. Combined with computer-simulated drug design and rapid determination of activity, researchers can quickly and efficiently modify the directional structure of natural compounds. In the structural modification of natural products, the application of amino acids can improve many properties. Therefore, amino acids have great application prospects in the structural modification of natural products.

## Author Contributions

QX and HD conceptualized the project. QX, HD, and XL contributed to the development and writing of the manuscript. XL and Z-SQ contributed in validating, reviewing, and supervising the project. All authors contributed to the article and approved the submitted version.

## Conflict of Interest

The authors declare that the research was conducted in the absence of any commercial or financial relationships that could be construed as a potential conflict of interest.

## References

[B1] Amoussa A. M. Lagnika L. Bourjot M. Vonthron-Senecheau C. Sanni A. (2016). Triterpenoids from Acacia ataxacantha DC: antimicrobial and antioxidant activities. BMC Complement Altern. Med. 16:284. 10.1186/s12906-016-1266-y 27520306 PMC4983094

[B2] Anbharasi V. Cao N. Feng S. S. (2010). Doxorubicin conjugated to D-alpha-tocopheryl polyethylene glycol succinate and folic acid as a prodrug for targeted chemotherapy. J. Biomed. Mater Res. A 94, 730–743. 10.1002/jbm.a.32734 20225211

[B3] Baloch S. K. Ma L. Wang X. L. Shi J. Zhu Y. Wu F. Y.. (2015). Design, synthesis and mechanism of novel shikonin derivatives as potent anticancer agents. Rsc. Adv. 5, 31759–31767. 10.1039/C5RA01872B

[B4] Baltina L. (2003). Chemical modification of glycyrrhizic acid as a route to new bioactive compounds for medicine. Curr. Med. Chem. 10, 155–171. 10.2174/0929867033368538 12570715

[B5] Barnham K. J. Djuran M. I. Murdoch P. S. Ranford J. D. Sadler P. J. (1996). Ring-opened adducts of the anticancer drug carboplatin with sulfur amino acids. Inorg. Chem. 35, 1065–1072. 10.1021/ic950973d 11666286

[B6] Bentley R. (2000). Mycophenolic Acid: a one hundred year odyssey from antibiotic to immunosuppressant. Chem. Rev. 100, 3801–3826. 10.1021/cr990097b 11749328

[B7] Bi W. Bi Y. Gao X. Yan X. Zhang Y. Harris J.. (2016). Pharmacological protection of mitochondrial function mitigates acute limb ischemia/reperfusion injury. Bioorg. Med. Chem. Lett. 26, 4042–4051. 10.1016/j.bmcl.2016.06.079 27390069

[B8] Bi Y. Xu J. Wu X. Ye W. Yuan S. Zhang L. (2007). Synthesis and cytotoxic activity of 17-carboxylic acid modified 23-hydroxy betulinic acid ester derivatives. Bioorg. Med. Chem. Lett. 17, 1475–1478. 10.1016/j.bmcl.2006.09.096 17275295

[B9] Bi Y. Yang X. Zhang T. Liu Z. Zhang X. Lu J.. (2015). Design, synthesis, nitric oxide release and antibacterial evaluation of novel nitrated ocotillol-type derivatives. Eur. J. Med. Chem. 101, 71–80. 10.1016/j.ejmech.2015.06.021 26114813

[B10] Borthwick A. D. Davies D. E. Ertl P. F. Exall A. M. Haley T. M. Hart G. J.. (2003). Design and synthesis of pyrrolidine-5,5'-trans-lactams (5-oxo-hexahydropyrrolo[3,2-b]pyrroles) as novel mechanism-based inhibitors of human cytomegalovirus protease. 4. Antiviral activity and plasma stability. J. Med. Chem. 46, 4428–4449. 10.1021/jm030810w 14521407

[B11] Brady S. F. Pawluczyk J. M. Lumma P. K. Feng D. M. Wai J. M. Jones R.. (2002). Design and synthesis of a pro-drug of vinblastine targeted at treatment of prostate cancer with enhanced efficacy and reduced systemic toxicity. J. Med. Chem. 45, 4706–4715. 10.1021/jm020139f 12361397

[B12] Cai E. Guo S. Yang L. Han M. Xia J. Zhao Y.. (2018). Synthesis and antitumour activity of arctigenin amino acid ester derivatives against H22 hepatocellular carcinoma. Nat. Prod. Res. 32, 406–411. 10.1080/14786419.2017.1314279 28415847

[B13] Cai E. B. Yang L. M. Jia C. X. Zhang W. Y. Zhao Y. Li W.. (2016). The synthesis and evaluation of arctigenin amino acid ester derivatives. Chem. Pharm. Bull. 64, 1466–1473. 10.1248/cpb.c16-0429 27383415

[B14] Cao Y. K. Li H. J. Song Z. F. Li Y. Huai Q. Y. (2014). Synthesis and biological evaluation of novel curcuminoid derivatives. Molecules 19, 16349–16372. 10.3390/molecules191016349 25314599 PMC6271059

[B15] Carroux C. J. Moeker J. Motte J. Lopez M. Bornaghi L. F. Katneni K.. (2013). Synthesis of acylated glycoconjugates as templates to investigate *in vitro* biopharmaceutical properties. Bioorg. Med. Chem. Lett. 23, 455–459 10.1016/j.bmcl.2012.11.056 23245512

[B16] Carubelli V. Castrini A. I. Lazzarini V. Gheorghiade M. Metra M. Lombardi C. (2015). Amino acids and derivatives, new treatment of chronicheart failure? Heart Fail Rev. 20, 39–51. 10.1007/s10741-014-9436-9 24925377

[B17] Cha Y. F. Zhang S. Su H. Ou Y. Fu X. Z. Jiang F. J.. (2014). *L*-Amino acid carbamate prodrugs of scutellarin: synthesis, physiochemical property, Caco-2 cell permeability, and *in vitro* anti-oxidative activity. Med. Chem. Res. 24, 2238–2246. 10.1007/s00044-014-1286-4

[B18] Chattopadhyay D. Arunachalam G. Mandal A. B. Sur T. K. Mandal S. C. Bhattacharya S. K. (2002). Antimicrobial and anti-inflammatory activity of folklore: Mallotus peltatus leaf extract. J. Ethnopharmacol. 82, 229–237. 10.1016/S0378-8741(02)00165-4 12242000

[B19] Chen A. Weber I. T. Harrison R. W. Leis J. (2006). Identification of amino acids in HIV-1 and avian sarcoma virus integrase subsites required for specific recognition of the long terminal repeat Ends. J. Biol. Chem. 281, 4173–4182. 10.1074/jbc.M510628200 16298997 PMC2656937

[B20] Chen S. H. Wang X. L. Xie X. H. Zheng L. Z. Yao D. Wang D. P.. (2012). Comparative study of osteogenic potential of a composite scaffold incorporating either endogenous bone morphogenetic protein-2 or exogenous phytomolecule icaritin: an *in vitro* efficacy study. Acta Biomater. 8, 3128–3137. 10.1016/j.actbio.2012.04.030 22543006

[B21] Chen X. Cui L. Duan X. Ma B. Zhong D. (2006). Pharmacokinetics and metabolism of the flavonoid scutellarin in humans after a single oral administration. Drug Metab. Dispos. 34, 1345–1352. 10.1124/dmd.106.009779 16714374

[B22] Chen X. Yang L. Oppenheim J. J. Howard M. Z. (2002). Cellular pharmacology studies of shikonin derivatives. Phytother. Res. 16, 199–209. 10.1002/ptr.1100 12164262

[B23] Chen Z. Duan H. Wang M. Han L. Liu Y. Zhu Y.. (2015). Synthesis, cytotoxicity and haemolytic activity of Pulsatilla saponin A, D derivatives. Bioorg. Med. Chem. Lett. 25, 2550–2554. 10.1016/j.bmcl.2015.04.049 25958248

[B24] Choi Y. H. Baek J. H. Yoo M. A. Chung H. Y. Kim N. D. Kim K. W. (2000). Induction of apoptosis by ursolic acid through activation of caspases and down-regulation of c-IAPs in human prostate epithelial cells. Int. J. Oncol. 17, 565–571. 10.3892/ijo.17.3.565 10938399

[B25] Cincinelli R. Musso L. Dallavalle S. Artali R. Tinelli S. Colangelo D.. (2013). Design, modeling, synthesis and biological activity evaluation of camptothecin-linked platinum anticancer agents. Eur. J. Med. Chem. 63, 387–400. 10.1016/j.ejmech.2013.02.022 23517728

[B26] Criado M. Balsera B. Mulet J. Sala S. Sala F. de la Torre-Martinez R.. (2016). 1,3-diphenylpropan-1-ones as allosteric modulators of alpha7 nACh receptors with analgesic and antioxidant properties. Future Med. Chem. 8, 731–749. 10.4155/fmc-2015-0001 27161515

[B27] Csuk R. Schwarz S. Kluge R. Strohl D. (2011a). Improvement of the cytotoxicity and tumor selectivity of glycyrrhetinic acid by derivatization with bifunctional amino acids. Arch Pharm. 344, 505–513. 10.1002/ardp.201100030 21674592

[B28] Csuk R. Schwarz S. Siewert B. Kluge R. Strohl D. (2011b). Synthesis and antitumor activity of ring A modified glycyrrhetinic acid derivatives. Eur. J. Med. Chem. 46, 5356–5369. 10.1016/j.ejmech.2011.08.038 21959232

[B29] Cui X. R. Tsukada M. Suzuki N. Shimamura T. Gao L. Koyanagi J.. (2008). Comparison of the cytotoxic activities of naturally occurring hydroxyanthraquinones and hydroxynaphthoquinones. Eur. J. Med. Chem. 43, 1206–1215. 10.1016/j.ejmech.2007.08.009 17949858

[B30] Dang Z. Qian K. Ho P. Zhu L. Lee K. H. Huang L.. (2012). Synthesis of betulinic acid derivatives as entry inhibitors against HIV-1 and bevirimat-resistant HIV-1 variants. Bioorg. Med. Chem. Lett. 22, 5190–5194. 10.1016/j.bmcl.2012.06.080 22818973 PMC3426442

[B31] Deng H. Huang X. Jin C. Jin C. M. Quan Z. S. (2020). Synthesis, *in vitro* and *in vivo* biological evaluation of dihydroartemisinin derivatives with potential anti-*Toxoplasma gondii* agents. Bioorg. Chem. 94:103467. 10.1016/j.bioorg.2019.103467 31791681

[B32] Dodds H. M. Rivory L. P. (1999). The mechanism for the inhibition of acetylcholinesterases by irinotecan (CPT-11). Mol. Pharmacol. 56, 1346–1353. 10.1124/mol.56.6.1346 10570064

[B33] Drag-Zalesinska M. Kulbacka J. Saczko J. Wysocka T. Zabel M. Surowiak P.. (2009). Esters of betulin and betulinic acid with amino acids have improved water solubility and are selectively cytotoxic toward cancer cells. Bioorg. Med. Chem. Lett. 19, 4814–4817. 10.1016/j.bmcl.2009.06.046 19560351

[B34] Drag-Zalesinska M. Wysocka T. Borska S. Drag M. Poreba M. Choromanska A.. (2015). The new esters derivatives of betulin and betulinic acid in epidermoid squamous carcinoma treatment - *in vitro* studies. Biomed. Pharmacother. 72, 91–97. 10.1016/j.biopha.2015.04.003 26054680

[B35] Erba E. Pocar D. Rossi L. M. (1998). New esters of R-(+)-usnic acid. Farmaco 53, 718–720. 10.1016/S0014-827X(98)00113-X

[B36] Fallarero A. Skogman M. Kujala J. Rajaratnam M. Moreira V. M. Yli-Kauhaluoma J.. (2013). (+)-Dehydroabietic acid, an abietane-type diterpene, inhibits *Staphylococcus aureus* biofilms *in vitro*. Int. J. Mol. Sci. 14, 12054–12072. 10.3390/ijms140612054 23739682 PMC3709773

[B37] Fan Y. Weinstein J. N. Kohn K. W. Shi L. M. Pommier Y. (1998). Molecular modeling studies of the DNA-topoisomerase I ternary cleavable complex with camptothecin. J. Med. Chem. 41, 2216–2226. 10.1021/jm9605445 9632354

[B38] Fang J. Huang T. Xia M. Deng L. Hao X. Wang Y.. (2018). Design and synthesis of novel monoterpenoid indole alkaloid-like analogues and their antitumour activities *in vitro*. Org. Biomol. Chem. 16, 3026–3037. 10.1039/C8OB00677F 29634066

[B39] Felczak K. Vince R. Pankiewicz K. W. (2014). NAD-based inhibitors with anticancer potential. Bioorg. Med. Chem. Lett. 24, 332–336. 10.1016/j.bmcl.2013.11.005 24269162

[B40] Filali I. Jelassi A. Jannet H. B. (2019). New bioactive esters and phosphonates semisynthesized from (±)-vasicinone: an alkaloid isolated from peganum harmala. Nat. Prod. Comm. 14, 1–9. 10.1177/1934578X19893544

[B41] Foti R. S. Dickmann L. J. Davis J. A. Greene R. J. Hill J. J. Howard M. L.. (2008). Metabolism and related human risk factors for hepatic damage by usnic acid containing nutritional supplements. Xenobiotica 38, 264–280. 10.1080/00498250701802514 18274956

[B42] Francisco C. S. Rodrigues L. R. Cerqueira N. M. Oliveira-Campos A. M. Rodrigues L. M. (2012). Synthesis of novel benzofurocoumarin analogues and their anti-proliferative effect on human cancer cell lines. Eur. J. Med. Chem. 47, 370–376. 10.1016/j.ejmech.2011.11.005 22119152

[B43] Fu H. J. Zhou Y. R. Bao B. H. Jia M. X. Zhao Y. Zhang L.. (2014). Tryptophan hydroxylase 1 (Tph-1)-targeted bone anabolic agents for osteoporosis. J. Med. Chem. 57, 4692–4709. 10.1021/jm5002293 24844139

[B44] Fu X. Z. Zhang W. Wang Y. L. Lan Y. Y. Wang A. M. Zhou W.. (2011). Design, synthesis and anti-oxidative evaluation of L-amino acid prodrugs of scutellarein. Yao Xue Xue Bao 46, 548–555. 10.1007/s11606-010-1517-4 21800542

[B45] Fu Y. Mo H. Y. Gao W. Hong J. Y. Lu J. Li P.. (2014). Affinity selection-based two-dimensional chromatography coupled with high-performance liquid chromatography-mass spectrometry for discovering xanthine oxidase inhibitors from Radix Salviae Miltiorrhizae. Anal. Bioanal. Chem. 406, 4987–4995. 10.1007/s00216-014-7902-9 24866714

[B46] Galanty A. Koczurkiewicz P. Wnuk D. Paw M. Karnas E. Podolak I.. (2017). Usnic acid and atranorin exert selective cytostatic and anti-invasive effects on human prostate and melanoma cancer cells. Toxicol. in Vitro 40, 161–169. 10.1016/j.tiv.2017.01.008 28095330

[B47] Garcia E. S. Azambuja P. (2004). Lignoids in insects: chemical probes for the study of ecdysis, excretion and Trypanosoma cruzi-triatomine interactions. Toxicon 44, 431–440. 10.1016/j.toxicon.2004.05.007 15302525

[B48] Gobec M. Tomasic T. Stimac A. Frkanec R. Trontelj J. Anderluh M.. (2018). Discovery of nanomolar desmuramylpeptide agonists of the innate immune receptor Nucleotide-Binding Oligomerization Domain-Containing Protein 2 (NOD2) possessing immunostimulatory properties. J. Med. Chem. 61, 2707–2724. 10.1021/acs.jmedchem.7b01052 29543461

[B49] Guerrant W. Patil V. Canzoneri J. C. Yao L. P. Hood R. Oyelere A. K. (2013). Dual-acting histone deacetylase-topoisomerase I inhibitors. Bioorg. Med. Chem. Lett. 23, 3283–3287. 10.1016/j.bmcl.2013.03.108 23622981 PMC3657756

[B50] Guo H. Y. Jin C. M. Zhang H. M. Jin C. M. Shen Q. K. Quan Z. S. (2019). Synthesis and biological evaluation of (+)-usnic acid derivatives as potential anti-*Toxoplasma gondii* agents. J. Agric. Food Chem. 67, 9630–9642. 10.1021/acs.jafc.9b02173 31365255

[B51] Hamed A. Frese M. Elgaafary M. Syrovets T. Sewald N. Simmet T.. (2020). Synthesis of novel feruloyl dipeptides with proapoptotic potential against different cancer cell lines. Bioorg. Chem. 97:103678. 10.1016/j.bioorg.2020.103678 32120076

[B52] Han H. W. Qiu H. Y. Hu C. Sun W. X. Yang R. W. Qi J. L.. (2016). Design, synthesis and anti-cancer activity evaluation of podophyllotoxin-norcantharidin hybrid drugs. Bioorg. Med. Chem. Lett. 26, 3237–3242. 10.1016/j.bmcl.2016.05.063 27262599

[B53] Han X. Song J. Lian L. H. Yao Y. L. Shao D. Y. Fan Y.. (2018). Ginsenoside 25-OCH3-PPD promotes activity of LXRs to ameliorate P2X7R-Mediated NLRP3 inflammasome in the development of hepatic fibrosis. J. Agric. Food Chem. 66, 7023–7035. 10.1021/acs.jafc.8b01982 29929367

[B54] Hedstrom L. (2009). IMP dehydrogenase: structure, mechanism, and inhibition. Chem. Rev. 109, 2903–2928. 10.1021/cr900021w 19480389 PMC2737513

[B55] Helfenstein A. Vahermo M. Nawrot D. A. Demirci F. Iscan G. Krogerus S.. (2017). Antibacterial profiling of abietane-type diterpenoids. Bioorg. Med. Chem. 25, 132–137. 10.1016/j.bmc.2016.10.019 27793449

[B56] Honda S. Miura Y. Masuda T. Masuda A. (2016). Effective conversion of metmyoglobin to oxymyoglobin by cysteine-substituted polyphenols. J. Agric. Food Chem. 64, 806–811. 10.1021/acs.jafc.5b05511 26753907

[B57] Hu X. Bai Z. Qiao J. Li H. Xu S. Wang X.. (2019). Effective enmein-type mimics of clinical candidate HAO472: design, synthesis and biological evaluation. Eur. J. Med. Chem. 171, 169–179. 10.1016/j.ejmech.2019.03.046 30921757

[B58] Hu X. Gao C. Tan C. Zhang C. Zhang H. Li S.. (2011). Design and synthesis of N-phosphoryl peptide modified podophyllotoxin derivatives as potent anticancer agents. Protein Pept. Lett. 18, 1258–1264. 10.2174/092986611797642652 21787278

[B59] Hu X. Wang Y. Xue J. Han T. Jiao R. Li Z.. (2017). Design and synthesis of novel nitrogen mustard-evodiamine hybrids with selective antiproliferative activity. Bioorg. Med. Chem. Lett. 27, 4989–4993. 10.1016/j.bmcl.2017.10.014 29037951

[B60] Huang C. Q. Li W. Wu B. Chen W. M. Chen L. H. Mo G. W.. (2016). Pheretima aspergillum decoction suppresses inflammation and relieves asthma in a mouse model of bronchial asthma by NF-kappaB inhibition. J. Ethnopharmacol. 189, 22–30. 10.1016/j.jep.2016.05.028 27184188

[B61] Huang L. Ho P. Lee K. H. Chen C. H. (2006). Synthesis and anti-HIV activity of bi-functional betulinic acid derivatives. Bioorg. Med. Chem. 14, 2279–2289. 10.1016/j.bmc.2005.11.016 16314103

[B62] Huang R. Z. Jin L. Yao G. Y. Dai W. L. Huang X. C. Liao Z. X.. (2017). Synthesis and molecular docking study of novel alizarin derivatives containing phosphoryl amino acid moiety as potential antitumor agents. Med. Chem. Res. 26, 2363–2374. 10.1007/s00044-017-1938-2

[B63] Ikeda M. (2003). Amino acid production processes. Adv. Biochem. Eng. Biotechnol. 79, 1–35. 10.1007/3-540-45989-8_1 12523387

[B64] Imbert T. F. (1998). Discovery of podophyllotoxins. Biochimie 80, 207–222. 10.1016/S0300-9084(98)80004-7 9615861

[B65] Iwaszkiewicz-Grzes D. Cholewinski G. Kot-Wasik A. Trzonkowski P. Dzierzbicka K. (2013). Synthesis and biological activity of mycophenolic acid-amino acid derivatives. Eur. J. Med. Chem. 69, 863–871. 10.1016/j.ejmech.2013.09.026 24121309

[B66] Jeong Y. H. Chung S. Y. Han A. R. Sung M. K. Jang D. S. Lee J.. (2007). P-glycoprotein inhibitory activity of two phenolic compounds, (-)-syringaresinol and tricin from Sasa borealis. Chem. Biodivers. 4, 12–16. 10.1002/cbdv.200790001 17256728

[B67] Ji Q. Ge Z. Ge Z. Chen K. Wu H. Liu X.. (2016). Synthesis and biological evaluation of novel phosphoramidate derivatives of coumarin as chitin synthase inhibitors and antifungal agents. Eur. J. Med. Chem. 108, 166–176. 10.1016/j.ejmech.2015.11.027 26647304

[B68] Jia Y. Dong X. Zhou P. Liu X. Pan L. Xin H.. (2012). The synthesis and biological evaluation of novel Danshensu-cysteine analog conjugates as cardiovascular-protective agents. Eur. J. Med. Chem. 55, 176–187. 10.1016/j.ejmech.2012.07.016 22841280

[B69] Jia Z. Xu C. Shen J. Xia T. Yang J. He Y. (2015). The natural compound celastrol inhibits necroptosis and alleviates ulcerative colitis in mice. Int. Immunopharmacol. 29, 552–559. 10.1016/j.intimp.2015.09.029 26454701

[B70] Jiang F. J. Fu X. Z. Wang S. W. Huang Y. Zhou W. Wang A. M.. (2013). Synthesis and physiochemical property evaluation of carbamate derivatives of scutellarin methyl ester. Chin. Chem. Lett. 24, 338–340. 10.1016/j.cclet.2013.02.003

[B71] Jin X. Zheng C. J. Song M. X. Wu Y. Sun L. P. Li Y. J.. (2012). Synthesis and antimicrobial evaluation of *L*-phenylalanine-derived C5-substituted rhodanine and chalcone derivatives containing thiobarbituric acid or 2-thioxo-4-thiazolidinone. Eur. J. Med. Chem. 56, 203–209. 10.1016/j.ejmech.2012.08.026 22982124

[B72] Jing Y. Wang G. Ge Y. Xu M. Gong Z. (2015). Synthesis, anti-tumor and anti-angiogenic activity evaluations of asiatic Acid amino Acid derivatives. Molecules 20, 7309–7324. 10.3390/molecules20047309 25905607 PMC6272655

[B73] Kapil A. (1993). Piperine: a potent inhibitor of Leishmania donovani promastigotes *in vitro*. Planta Med. 59, 474. 10.1055/s-2006-959737 8255940

[B74] Kates S. A. Casale R. A. Baguisi A. Beeuwkes R. (2014). Lipoic acid analogs with enhanced pharmacological activity. Bioorg. Med. Chem. 22, 505–512. 10.1016/j.bmc.2013.10.057 24316353

[B75] Kawaguchi T. Izumi N. Charlton M. R. Sata M. (2011). Branched-chain amino acids as pharmacological nutrients in chronic liver disease. Hepatology 54, 1063–1070. 10.1002/hep.24412 21563202

[B76] Kim M. K. Chong Y. (2013). Design, synthesis, and biological evaluation of resveratrol derivatives as PPAR alpha agonists. J. Korean Soc. Applied Biol. Chem. 56, 353–356. 10.1007/s13765-013-3086-9

[B77] Kim M. K. Choo H. Chong Y. (2014). Water-soluble and cleavable quercetin-amino acid conjugates as safe modulators for P-glycoprotein-based multidrug resistance. J. Med. Chem. 57, 7216–7233. 10.1021/jm500290c 25122155

[B78] Kim M. K. Kim Y. Choo H. Chong Y. (2017). Quercetin-glutamic acid conjugate with a non-hydrolysable linker; a novel scaffold for multidrug resistance reversal agents through inhibition of P-glycoprotein. Bioorg. Med. Chem. 25, 1219–1226. 10.1016/j.bmc.2016.12.034 28043777

[B79] Kim M. K. Park K. S. Yeo W. S. Choo H. Chong Y. (2009). *In vitro* solubility, stability and permeability of novel quercetin-amino acid conjugates. Bioorg. Med. Chem. 17, 1164–1171. 10.1016/j.bmc.2008.12.043 19128975

[B80] Kostakis C. Byard R. W. (2009). Sudden death associated with intravenous injection of toad extract. Forensic. Sci. Int. 188, 1–5. 10.1016/j.forsciint.2009.02.006 19303230

[B81] Kotha R. R. Luthria D. L. (2019). Curcumin: biological, pharmaceutical, nutraceutical, and analytical aspects. Molecules 24::2930. 10.3390/molecules24162930 31412624 PMC6720683

[B82] Lan P. Wang J. Zhang D. M. Shu C. Cao H. H. Sun P. H.. (2011). Synthesis and antiproliferative evaluation of 23-hydroxybetulinic acid derivatives. Eur. J. Med. Chem. 46, 2490–2502. 10.1016/j.ejmech.2011.03.038 21496972

[B83] LaVail J. H. Tauscher A. N. Aghaian E. Harrabi O. Sidhu S. S. (2003). Axonal transport and sorting of herpes simplex virus components in a mature mouse visual system. J. Virol. 77, 6117–6126. 10.1128/JVI.77.11.6117-6126.2003 12743269 PMC155024

[B84] Lebeaux D. Chauhan A. Rendueles O. Beloin C. (2013). From *in vitro* to *in vivo* models of bacterial biofilm-related infections. Pathogens. 2, 288–356. 10.3390/pathogens2020288 25437038 PMC4235718

[B85] Lee C. C. Huang K. F. Chang D. M. Hsu J. J. Huang F. C. Shih K. N.. (2012). Design, synthesis and evaluation of telomerase inhibitory, hTERT repressing, and anti-proliferation activities of symmetrical 1,8-disubstituted amidoanthraquinones. Eur. J. Med. Chem. 50, 102–112. 10.1016/j.ejmech.2012.01.044 22357112

[B86] Lee K. H. (2004). Current developments in the discovery and design of new drug candidates from plant natural product leads. J. Nat. Prod. 67, 273–283. 10.1021/np030373o 14987069

[B87] Li L. Liu W. Y. Feng F. Wu C. Y. Xie N. (2013). Synthesis and *in vitro* cytotoxicity evaluation of baicalein amino acid derivatives. Chin. J. Nat. Med. 11, 284–288. 10.3724/SP.J.1009.2013.00284 23725843

[B88] Li X. Zhang X. Sun H. Zhang L. Gao Y. Wang J.. (2012). Synthesis and anti-tumor evaluation of novel C-37 modified derivatives of gambogic acid. Chin. J. Chem. 30, 1083–1091. 10.1002/cjoc.201100693

[B89] Li Y. Q. Yang F. Wang L. Cao Z. Han T. J. Duan Z. A.. (2016). Phosphoramidate protides of five flavones and their antiproliferative activity against HepG2 and L-O2 cell lines. Eur. J. Med. Chem. 112, 196–208. 10.1016/j.ejmech.2016.02.012 26896708

[B90] Li Z. Shen M. Z. Li W. (2017). Design, synthesis and druggability evaluation of liguzinediol valine ester prodrug as promissing inotropic agent. J. Nanjing Univ. Tradit Chin. Med. 33, 412–416. 10.14148/j.issn.1672-0482.2017.0412

[B91] Lin H. Y. Chen W. Shi J. Kong W. Y. Qi J. L. Wang X. M.. (2013). Design, synthesis and biological evaluation of cinnamic acyl shikonin derivatives. Chem. Biol. Drug Des. 81, 275–283. 10.1111/cbdd.12077 23066914

[B92] Lin H. Y. Li Z. K. Bai L. F. Baloch S. K. Wang F. Qiu H. Y.. (2015). Synthesis of aryl dihydrothiazol acyl shikonin ester derivatives as anticancer agents through microtubule stabilization. Biochem. Pharmacol. 96, 93–106. 10.1016/j.bcp.2015.04.021 25957661

[B93] Lin L. L. Liu A. J. Liu J. G. Yu X. H. Qin L. P. Su D. F. (2007). Protective effects of scutellarin and breviscapine on brain and heart ischemia in rats. J. Cardiovasc. Pharmacol. 50, 327–332. 10.1097/FJC.0b013e3180cbd0e7 17878763

[B94] Lin S. Koh J. J. Aung T. T. Sin W. L. W. Lim F. Wang L.. (2017). Semisynthetic flavone-derived antimicrobials with therapeutic potential against Methicillin-Resistant *Staphylococcus aureus* (MRSA). J. Med. Chem. 60, 6152–6165. 10.1021/acs.jmedchem.7b00380 28636355

[B95] Liu X. Niu H. Li Q. Gu P. (2019). Metabolic engineering for the production of l-phenylalanine in *Escherichia coli*. 3 Biotech 9:85. 10.1007/s13205-019-1619-6 30800596 PMC6385074

[B96] Liu X. K. Ye B. J. Wu Y. Lin Z. H. Zhao Y. Q. Piao H. R. (2011). Synthesis and anti-tumor evaluation of panaxadiol derivatives. Eur. J. Med. Chem. 46, 1997–2002. 10.1016/j.ejmech.2011.02.022 21439693

[B97] Liu Z. Xiao M. Waters J. Velazquez O. C. (2009). QS248. a novel approach in regulating neovascularization: targeting stromal fibroblasts by modulation of Notch1 signaling. J. Surg. Res. 151, 293–293. 10.1016/j.jss.2008.11.551

[B98] Lo Y. L. Ho C. T. Tsai F. L. (2008). Inhibit multidrug resistance and induce apoptosis by using glycocholic acid and epirubicin. Eur. J. Pharm. Sci. 35, 52–67. 10.1016/j.ejps.2008.06.003 18606222

[B99] Ma C. M. Kully M. Khan J. K. Hattori M. Daneshtalab M. (2007). Synthesis of chlorogenic acid derivatives with promising antifungal activity. Bioorg. Med. Chem. 15, 6830–6833. 10.1016/j.bmc.2007.07.038 17761420

[B100] Ma C. M. Nakamura N. Hattori M. Kakuda H. Qiao J. C. Yu H. L. (2000). Inhibitory effects on HIV-1 protease of constituents from the wood of Xanthoceras sorbifolia. J. Nat. Prod. 63, 238–242. 10.1021/np9902441 10691716

[B101] Malysheva Y. B. Voitovich Y. V. Sharonova E. A. Combes S. Svirshchevskaya E. V. Vodovozova E. L.. (2014). Novel water-soluble anticancer agents derived from 4-arylcoumarins. Russ. Chem. Bull. 62, 1103–1110. 10.1007/s11172-013-0149-3

[B102] Manner S. Vahermo M. Skogman M. E. Krogerus S. Vuorela P. M. Yli-Kauhaluoma J.. (2015). New derivatives of dehydroabietic acid target planktonic and biofilm bacteria in *Staphylococcus aureus* and effectively disrupt bacterial membrane integrity. Eur. J. Med. Chem. 102, 68–79. 10.1016/j.ejmech.2015.07.038 26241878

[B103] Masuda T. Inai M. Miura Y. Masuda A. Yamauchi S. (2013). Effect of polyphenols on oxymyoglobin oxidation: prooxidant activity of polyphenols *in vitro* and inhibition by amino acids. J. Agric. Food Chem. 61, 1097–1104. 10.1021/jf304775x 23311772

[B104] Meng G. Li J. Wang G. Dong M. Zhang Q. (2014). Synthesis and cytotoxic activities of the amino acid-conjugates of 10-Hydroxycamptothecin. Chin. J. Org. Chem. 34, 155–160. 10.6023/cjoc201307049Z

[B105] Mook-Jung I. Shin J. E. Yun S. H. Huh K. Koh J. Y. Park H. K.. (1999). Protective effects of asiaticoside derivatives against beta-amyloid neurotoxicity. J. Neurosci. Res. 58, 417–425. 10.1002/(SICI)1097-4547(19991101)58:3<417::AID-JNR7>3.0.CO;2-G 10518115

[B106] Moscatelli V. Hnatyszyn O. Acevedo C. Megias J. Alcaraz M. J. Ferraro G. (2006). Flavonoids from Artemisia copa with anti-inflammatory activity. Planta Med. 72, 72–74. 10.1055/s-2005-873177 16450301

[B107] Nicolaou K. C. Pfefferkorn J. A. Roecker A. J. Cao G. Q. Barluenga S. Mitchell H. J. (2000). Natural product-like combinatorial libraries based on privileged structures. 1. general principles and solid-phase synthesis of benzopyrans. J. Amer. Chem. Soc. 122, 9939–9953. 10.1021/ja002033k

[B108] Nitz K. Lacy M. Atzler D. (2019). Amino acids and their metabolism in atherosclerosis. Arterioscler. Thromb. Vasc. Bio. 39, 319–330. 10.1161/ATVBAHA.118.311572 30650999

[B109] Pang C. Luo J. Liu C. Wu X. Wang D. (2018). Synthesis and biological evaluation of a series of novel celastrol derivatives with amino acid chain. Chem. Biodivers. 15, 1–11. 10.1002/cbdv.201800059 29633499

[B110] Paris D. Ganey N. J. Laporte V. Patel N. S. Beaulieu-Abdelahad D. Bachmeier C.. (2010). Reduction of beta-amyloid pathology by celastrol in a transgenic mouse model of Alzheimer's disease. J. Neuroinflam. 7:17. 10.1186/1742-2094-7-17 20211007 PMC2841120

[B111] Park B. C. Bosire K. O. Lee E. S. Lee Y. S. Kim J. A. (2005). Asiatic acid induces apoptosis in SK-MEL-2 human melanoma cells. Cancer Lett. 218, 81–90. 10.1016/j.canlet.2004.06.039 15639343

[B112] Patagar D. Kusanur R. Sitwala N. D. Ghate M. D. Saravanakumar S. Nembenna S.. (2019). Synthesis of novel 4-substituted coumarins, docking studies, and DHODH inhibitory activity. J. Heterocyclic Chem. 56, 2761–2771. 10.1002/jhet.3644

[B113] Pecere T. Gazzola M. V. Mucignat C. Parolin C. Palu G. (2000). Aloe-emodin is a new type of anticancer agent with selective activity against neuroectodermal tumors1. Cancer Res. 60, 2800–2804. 10850417

[B114] Piska K. Galanty A. Koczurkiewicz P. Zmudzki P. Potaczek J. Podolak I.. (2018). Usnic acid reactive metabolites formation in human, rat, and mice microsomes. implication for hepatotoxicity. Food Chem. Toxicol. 120, 112–118. 10.1016/j.fct.2018.07.005 29981369

[B115] Pu X. Zhang C. R. Gao H. C. Gao Y. J. Huang L. Zhu L.. (2020). Biosynthesis-inspired mining and identification of untapped alkaloids in Camptotheca acuminate for enzyme discovery using ultra-high performance liquid chromatography coupled with quadrupole-time of flight-mass spectrometry. J. Chromatogr. A 1620:461036. 10.1016/j.chroma.2020.461036 32201039

[B116] Pugazhenthi S. Akhov L. Selvaraj G. Wang M. Alam J. (2007). Regulation of heme oxygenase-1 expression by demethoxy curcuminoids through Nrf2 by a PI3-kinase/Akt-mediated pathway in mouse beta-cells. Am. J. Physiol. Endocrinol. Metab. 293, 645–655. 10.1152/ajpendo.00111.2007 17535857

[B117] Qian K. Kim S. Y. Hung H. Y. Huang L. Chen C. H. Lee K. H. (2011). New betulinic acid derivatives as potent proteasome inhibitors. Bioorg. Med. Chem. Lett. 21, 5944–5947. 10.1016/j.bmcl.2011.07.072 21856154 PMC3171619

[B118] Qu F. Zhao C. Liu Y. Cao J. Li W. Zhao Y. (2015). Semi-synthesis and anti-tumor evaluation of novel 25-hydroxyprotopanaxadiol derivatives as apoptosis inducing agents. Med. Chem. Comm. 6, 2004–2011. 10.1039/C5MD00382B

[B119] Ren Q. Yang G. Guo M. Guo J. Li Y. Lu J.. (2019). Design, synthesis, and discovery of ocotillol-type amide derivatives as orally available modulators of P-glycoprotein-mediated multidrug resistance. Eur. J. Med. Chem. 161, 118–130. 10.1016/j.ejmech.2018.10.038 30347326

[B120] Rodrigues T. Reker D. Schneider P. Schneider G. (2016). Counting on natural products for drug design. Nat. Chem. 8, 531–541. 10.1038/nchem.2479 27219696

[B121] Salvador J. A. R. Leal A. S. Valdeira A. S. Goncalves B. M. F. Alho D. P. S. Figueiredo S. A. C.. (2017). Oleanane-, ursane-, and quinone methide friedelane-type triterpenoid derivatives: recent advances in cancer treatment. Eur. J. Med. Chem. 142, 95–130. 10.1016/j.ejmech.2017.07.013 28754470

[B122] Sarciron M. E. Saccharin C. Petavy A. F. Peyron F. (2000). Effects of artesunate, dihydroartemisinin, and an artesunate-dihydroartemisinin combination against *Toxoplasma gondii*. Am. J. Trop Med. Hyg. 62, 73–76. 10.4269/ajtmh.2000.62.73 10761727

[B123] Schwarz S. Csuk R. (2010). Synthesis and antitumour activity of glycyrrhetinic acid derivatives. Bioorg. Med. Chem. 18, 7458–7474. 10.1016/j.bmc.2010.08.054 20932766

[B124] Schwarz S. Siewert B. Xavier N. M. Jesus A. R. Rauter A. P. Csuk R. (2014). A “natural” approach: synthesis and cytoxicity of monodesmosidic glycyrrhetinic acid glycosides. Eur. J. Med. Chem. 72, 78–83. 10.1016/j.ejmech.2013.11.024 24361520

[B125] Shao J. W. Dai Y. C. Xue J. P. Wang J. C. Lin F. P. Guo Y. H. (2011). *In vitro* and *in vivo* anticancer activity evaluation of ursolic acid derivatives. Eur. J. Med. Chem. 46, 2652–2661. 10.1016/j.ejmech.2011.03.050 21514015

[B126] Shen S. D. Zhang G. P. Lei M. Hua L. H. (2012). First total synthesis of salvianolic acid C, tournefolic acid A, and tournefolal. Arkivoc 6, 204–213. 10.3998/ark.5550190.0013.619

[B127] Shi M. Sun J. Zhou J. Yu H. Yu S. Xia G.. (2018). Phase I dose escalation and pharmacokinetic study on the nanoparticle formulation of polymeric micellar paclitaxel for injection in patients with advanced solid malignancies. Invest. New Drugs 36, 269–277. 10.1007/s10637-017-0506-4 28868573

[B128] Shi Q. W. Li L. G. Huo C. H. Zhang M. L. Wang Y. F. (2010). Study on natural medicinal chemistry and new drug development. Chin. Trad. Herbal Drugs 41, 1583–1589.

[B129] Siebert A. Wysocka M. Krawczyk B. Cholewinski G. Rachon J. (2018). Synthesis and antimicrobial activity of amino acid and peptide derivatives of mycophenolic acid. Eur. J. Med. Chem. 143, 646–655. 10.1016/j.ejmech.2017.11.094 29216563 PMC7173178

[B130] Singh I. P. Jain S. K. Kaur A. Singh S. Kumar R. Garg P.. (2010). Synthesis and antileishmanial activity of piperoyl-amino acid conjugates. Eur. J. Med. Chem. 45, 3439–3445. 10.1016/j.ejmech.2010.04.033 20546981

[B131] Smith P. F. Ogundele A. Forrest A. Wilton J. Salzwedel K. Doto J.. (2007). Phase I and II study of the safety, virologic effect, and pharmacokinetics/pharmacodynamics of single-dose 3-o-(3',3'-dimethylsuccinyl)betulinic acid (bevirimat) against human immunodeficiency virus infection. Antimicrob. Agents Chemother. 51, 3574–3581. 10.1128/AAC.00152-07 17638699 PMC2043264

[B132] Song M. X. Deng X. Q. Li Y. R. Zheng C. J. Hong L. Piao H. R. (2014). Synthesis and biological evaluation of (E)-1-(substituted)-3-phenylprop-2-en-1-ones bearing rhodanines as potent anti-microbial agents. J. Enzyme Inhib. Med. Chem. 29, 647–653. 10.3109/14756366.2013.837899 24102526

[B133] Song X. Liu Y. Ma J. He J. Zheng X. Lei X.. (2014). Synthesis of novel amino acid derivatives containing chrysin as anti-tumor agents against human gastric carcinoma MGC-803 cells. Med. Chem. Res. 24, 1789–1798. 10.1007/s00044-014-1267-7

[B134] Sowunmi A. Akano K. Ayede A. I. Ntadom G. Aderoyeje T. Adewoye E. O.. (2016). Clinical illness and outcomes in Nigerian children with late-appearing anaemia after artemisinin-based combination treatments of uncomplicated falciparum malaria. BMC Infect. Dis. 16:240. 10.1186/s12879-016-1565-4 27246468 PMC4888541

[B135] Sun P. Wu G. Qiu Z. Chen Y. (2014). Preparation of L-alanine-(14-oridonin) Ester Trifluoroacetate for Treatment of Cancer. Jiangsu Hengrui Medicine Co., Ltd., Peop. *Rep. China*, 10.

[B136] Tang H. J. Zhang X. W. Yang L. Li W. Li J. H. Wang J. X.. (2016). Synthesis and evaluation of xanthine oxidase inhibitory and antioxidant activities of 2-arylbenzo[b]furan derivatives based on salvianolic acid C. Eur. J. Med. Chem. 124, 637–648. 10.1016/j.ejmech.2016.08.019 27614410

[B137] Tatsuzaki J. Taniguchi M. Bastow K. F. Nakagawa-Goto K. Morris-Natschke S. L. Itokawa H.. (2007). Anti-tumor agents 255: novel glycyrrhetinic acid-dehydrozingerone conjugates as cytotoxic agents. Bioorg. Med. Chem. 15, 6193–6199. 10.1016/j.bmc.2007.06.027 17591444 PMC2034305

[B138] Thimmegowda N. R. Park C. Shwetha B. Sakchaisri K. Liu K. Hwang J.. (2015). Synthesis and antitumor activity of natural compound aloe emodin derivatives. Chem. Biol. Drug Des. 85, 638–644. 10.1111/cbdd.12448 25323822

[B139] Ukiya M. Kawaguchi T. Ishii K. Ogihara E. Tachi Y. Kurita M.. (2013). Cytotoxic activities of amino acid-conjugate derivatives of abietane-type diterpenoids against human cancer cell lines. Chem. Biodivers. 10, 1260–1268. 10.1002/cbdv.201300043 23847070

[B140] Verghese J. Nguyen T. Oppegard L. M. Seivert L. M. Hiasa H. Ellis K. C. (2013). Flavone-based analogues inspired by the natural product simocyclinone D8 as DNA gyrase inhibitors. Bioorg. Med. Chem. Lett. 23, 5874–5877. 10.1016/j.bmcl.2013.08.094 24060488

[B141] Wang J. Hu X. L. Wen W. H. Yang L. L. Zhu Y. L. (2012). Synthesis and activity of 3-Amino acid derivatives of glycyrrhetinic acid. Chin. J. Appl. Chem. 29, 873–877. 10.3724/SP.J.1095.2012.0037120851614

[B142] Wang K. Y. Zhou Z. W. Zhang H. Y. Cao Y. C. Xu J. Y. Ma C.. (2018). Design, Synthesis and Antibacterial Evaluation of 3-Substituted Ocotillol-Type Derivatives. Molecules 23, 87–101. 10.3390/molecules23123320 30558186 PMC6321515

[B143] Wang L. Li D. Xu S. Cai H. Yao H. Zhang Y.. (2012). The conversion of oridonin to spirolactone-type or enmein-type diterpenoid: synthesis and biological evaluation of ent-6,7-seco-oridonin derivatives as novel potential anticancer agents. Eur. J. Med. Chem. 52, 242–250. 10.1016/j.ejmech.2012.03.024 22483090

[B144] Wang P. Bi X. L. Xu J. Yuan H. N. Piao H. R. Zhao Y. Q. (2013). Synthesis and anti-tumor evaluation of novel 25-hydroxyprotopanaxadiol analogs incorporating natural amino acids. Steroids 78, 203–209. 10.1016/j.steroids.2012.09.012 23178255

[B145] Wang S. R. Fang W. S. (2009). Pentacyclic triterpenoids and their saponins with apoptosis-inducing activity. Curr. Topics Med. Chem. 9, 1581–1596. 10.2174/156802609789909821 19903161

[B146] Wen T. Xu W. Liang L. Li J. Ding X. Chen X.. (2015). Clinical efficacy of andrographolide sulfonate in the treatment of severe Hand, Foot, and Mouth Disease (HFMD) is dependent upon inhibition of neutrophil activation. Phytoth. Res. 29, 1161–1167. 10.1002/ptr.5361 25960284

[B147] Whiteman M. Tritschler H. Halliwell B. (1996). Protection against peroxynitrite-dependent tyrosine nitration and α1-antiproteinase inactivation by oxidized and reduced lipoic acid. FEBS Lett. 379, 74–76. 10.1016/0014-5793(95)01489-6 8566234

[B148] Wu G. (2009). Amino acids: metabolism, functions, and nutrition. Amino Acids 37, 1–17. 10.1007/s00726-009-0269-0 19301095

[B149] Wu G. R. Xu B. Yang Y. Q. Zhang X. Y. Fang K. Ma T.. (2018). Synthesis and biological evaluation of podophyllotoxin derivatives as selective antitumor agents. Eur. J. Med. Chem. 155, 183–196. 10.1016/j.ejmech.2018.05.052 29886322

[B150] Wu Y. L. Wan Y. Jin X. J. OuYang B. Q. Bai T. Zhao Y. Q.. (2011). 25-OCH_3_-PPD induces the apoptosis of activated t-HSC/Cl-6 cells via c-FLIP-mediated NF-kappaB activation. Chem. Biol. Interact. 194, 106–112. 10.1016/j.cbi.2011.08.010 21924252

[B151] Wu Y. Z. Cao D. Wang F. Ma L. Gao G. Chen L. J. (2015). Synthesis and evaluation of millepachine amino acid prodrugs with enhanced solubility as antitumor agents. Chem. Bio. Drug Des. 86, 559–567. 10.1111/cbdd.12507 25643726

[B152] Yang C. J. Song Z. L. Goto M. Hsu P. L. Zhang X. S. Yang Q. R.. (2017). Design, semisynthesis and potent cytotoxic activity of novel 10-fluorocamptothecin derivatives. Bioorg. Med. Chem. Lett. 27, 4694–4697. 10.1016/j.bmcl.2017.09.012 28927790 PMC5831374

[B153] Ye D. Shi Q. Leung C. H. Kim S. W. Park S. Y. Gullen E. A.. (2012). Antitumor agents 294. novel E-ring-modified camptothecin-4beta-anilino-4'-O-demethyl-epipodophyllotoxin conjugates as DNA topoisomerase I inhibitors and cytotoxic agents. Bioorg. Med. Chem. 20, 4489–4494. 10.1016/j.bmc.2012.05.030 22698783 PMC3389137

[B154] You L. Zhao M. Regenstein J. M. Ren J. (2011). *In vitro* antioxidant activity and *in vivo* anti-fatigue effect of loach (Misgurnus anguillicaudatus) peptides prepared by papain digestion. Food Chem. 124, 188–194. 10.1016/j.foodchem.2010.06.007 21675717

[B155] Yu H. Jin H. Gong W. Wang Z. Liang H. (2013). Pharmacological actions of multi-target-directed evodiamine. Molecules 18, 1826–1843. 10.3390/molecules18021826 23434865 PMC6270287

[B156] Yu X. M. Ramiandrasoa F. Guetzoyan L. Pradines B. Quintino E. Gadelle D.. (2012). Synthesis and biological evaluation of acridine derivatives as antimalarial agents. Chem. Med. Chem. 7, 587–605. 10.1002/cmdc.201100554 22331612

[B157] Yuan W. Guo J. Wang X. Su G. Zhao Y. (2018). Non-protein amino acid derivatives of 25-methoxylprotopanaxadiol/25-hydroxyprotopanaxadioland their anti-tumour activity evaluation. Steroids 129, 1–8. 10.1016/j.steroids.2017.11.003 29129719

[B158] Zhang C. Li X. Gao Y. Zhang L. Fu X. (2007). Synthesis and primary research on antitumor activity of three new panaxadiol fatty acid esters. Chem. Res. Chin. Univ. 23, 176–182. 10.1016/S1005-9040(07)60037-3

[B159] Zhang G. Ye X. Ji D. Zhang H. Sun F. Shang C.. (2014). Inhibition of lung tumor growth by targeting EGFR/VEGFR-Akt/NF-kappaB pathways with novel theanine derivatives. Oncotarget 5, 8528–8543. 10.18632/oncotarget.2336 25138052 PMC4226702

[B160] Zhang H. B. Shen Q. K. Wang H. Jin C. M. Jin C. M. Quan Z. S. (2018). Synthesis and evaluation of novel arctigenin derivatives as potential anti-*Toxoplasma gondii* agents. Eur. J. Med. Chem. 158, 414–427. 10.1016/j.ejmech.2018.08.087 30237124

[B161] Zhang H. J. Zhang G. R. Piao H. R. Quan Z. S. (2017). Synthesis and characterisation of celastrol derivatives as potential anticancer agents. J. Enzyme Inhibit. Med. Chem. 33, 190–198. 10.1080/14756366.2017.1404590 29231066 PMC6009949

[B162] Zhang L. J. Chen L. Lu Y. Wu J. M. Xu B. Sun Z. G.. (2010). Danshensu has anti-tumor activity in B16F10 melanoma by inhibiting angiogenesis and tumor cell invasion. Eur. J. Pharmacol. 643, 195–201. 10.1016/j.ejphar.2010.06.045 20621088

[B163] Zhang T. J. Li S. Y. Yuan W. Y. Zhang Y. Meng F. H. (2018). Design, synthesis, and molecular docking studies of N-(9,10-anthraquinone-2-carbonyl)amino acid derivatives as xanthine oxidase inhibitors. Chem. Biol. Drug Des. 91, 893–901. 10.1111/cbdd.13156 29197158

[B164] Zhang Y. N. Zhang W. Hong D. Shi L. Shen Q. Li J. Y.. (2008). Oleanolic acid and its derivatives: new inhibitor of protein tyrosine phosphatase 1B with cellular activities. Bioorg. Med. Chem. 16, 8697–8705. 10.1016/j.bmc.2008.07.080 18707891

[B165] Zhao H. Guo Y. Li S. Han R. Ying J. Zhu H.. (2015). A novel anti-cancer agent Icaritin suppresses hepatocellular carcinoma initiation and malignant growth through the IL-6/Jak2/Stat3 pathway. Oncotarget 6, 31927–31943. 10.18632/oncotarget.5578 26376676 PMC4741651

[B166] Zheng L. Fu Y. Zhuang L. Gai R. Ma J. Lou J.. (2014). Simultaneous NF-kappaB inhibition and E-cadherin upregulation mediate mutually synergistic anticancer activity of celastrol and SAHA *in vitro* and *in vivo*. Int. J. Cancer 135, 1721–1732. 10.1002/ijc.28810 24615207

[B167] Zhou F. Wu G. R. Cai D. S. Xu B. Yan M. M. Ma T.. (2019). Synthesis and biological activity of glycyrrhetinic acid derivatives as antitumor agents. Eur. J. Med. Chem. 178, 623–635. 10.1016/j.ejmech.2019.06.029 31226654

[B168] Zhou M. Liu M. He X. Yu H. Wu D. Yao Y.. (2014). Synthesis and biological evaluation of novel 10-substituted-7-ethyl-10-hydroxycamptothecin (SN-38) prodrugs. Molecules 19, 19718–19731. 10.3390/molecules191219718 25438082 PMC6270839

[B169] Zhou Z. Y. Zhao W. R. Shi W. T. Xiao Y. Ma Z. L. Xue J. G.. (2019). Endothelial-dependent and independent vascular relaxation effect of tetrahydropalmatine on rat aorta. Front. Pharmacol. 10:336. 10.3389/fphar.2019.00336 31057398 PMC6477965

[B170] Zhu P. Bi Y. Xu J. Li Z. Liu J. Zhang L.. (2009). Terpenoids. III: synthesis and biological evaluation of 23-hydroxybetulinic acid derivatives as novel inhibitors of glycogen phosphorylase. Bioorg. Med. Chem. Lett. 19, 6966–6969. 10.1016/j.bmcl.2009.10.055 19889536

[B171] Zhu Q. Yu X. Shen Q. Zhang Q. Su M. Zhou Y.. (2018). A series of camptothecin prodrugs exhibit HDAC inhibition activity. Bioorg. Med. Chem. 26, 4706–4715. 10.1016/j.bmc.2018.08.008 30115492

[B172] Zhuang C. Zhang W. Sheng C. Zhang W. Xing C. Miao Z. (2017). Chalcone: a privileged structure in medicinal chem. Chem. Rev. 117, 7762–7810. 10.1021/acs.chemrev.7b00020 28488435 PMC6131713

